# BCL11B Regulates Epithelial Proliferation and Asymmetric Development of the Mouse Mandibular Incisor

**DOI:** 10.1371/journal.pone.0037670

**Published:** 2012-05-22

**Authors:** Kateryna Kyrylkova, Sergiy Kyryachenko, Brian Biehs, Ophir Klein, Chrissa Kioussi, Mark Leid

**Affiliations:** 1 Department of Pharmaceutical Sciences, College of Pharmacy, Oregon State University, Corvallis, Oregon, United States of America; 2 Departments of Orofacial Sciences and Pediatrics and Program in Craniofacial and Mesenchymal Biology, University of California San Francisco San, Francisco California, United States of America; University of Southern California, United States of America

## Abstract

Mouse incisors grow continuously throughout life with enamel deposition uniquely on the outer, or labial, side of the tooth. Asymmetric enamel deposition is due to the presence of enamel-secreting ameloblasts exclusively within the labial epithelium of the incisor. We have previously shown that mice lacking the transcription factor BCL11B/CTIP2 (BCL11B hereafter) exhibit severely disrupted ameloblast formation in the developing incisor. We now report that BCL11B is a key factor controlling epithelial proliferation and overall developmental asymmetry of the mouse incisor: BCL11B is necessary for proliferation of the labial epithelium and development of the epithelial stem cell niche, which gives rise to ameloblasts; conversely, BCL11B suppresses epithelial proliferation, and development of stem cells and ameloblasts on the inner, or lingual, side of the incisor. This bidirectional action of BCL11B in the incisor epithelia appears responsible for the asymmetry of ameloblast localization in developing incisor. Underlying these spatio-specific functions of BCL11B in incisor development is the regulation of a large gene network comprised of genes encoding several members of the FGF and TGFβ superfamilies, Sprouty proteins, and Sonic hedgehog. Our data integrate BCL11B into these pathways during incisor development and reveal the molecular mechanisms that underlie phenotypes of both *Bcl11b^−/−^* and Sprouty mutant mice.

## Introduction

Tooth initiation in the mouse is characterized by a thickening of the oral epithelium at embryonic day (E) 11.5. The proliferating epithelium invaginates into the underlying neural crest-derived mesenchyme and forms a bud at E12.5–E13.5 (bud stage). The epithelium expands and folds around the condensed mesenchyme to form a cap-like structure at E14.5 (cap stage). The cap stage is characterized by formation of the enamel knot, a critical signaling center, and lateral protrusions of the epithelium, known as cervical loops (CLs). CLs extend during bell stage (E16.5–E18.5), at which point cytodifferentiation begins [1], [2], [3], [4].

Continuous growth of the rodent incisor requires the presence of epithelial and mesenchymal stem cells that provide a continuous supply of enamel-producing ameloblasts and dentin-producing odontoblasts, respectively. Epithelial stem cells (EpSCs) are slow-cycling cells located in the CLs [5], [6], [7]. The labial CL consists of a core stellate reticulum (SR) and stratum intermedium cells surrounded by basal epithelial cells, known as the inner and outer enamel epithelium (IEE and OEE, respectively) [8]. EpSCs reside in the labial CL and give rise to transit amplifying cells that migrate anteriorly along the IEE while sequentially differentiating to mitotic pre-ameloblasts, post-mitotic secretory ameloblasts, and mature ameloblasts [9]. The lingual CL contains a smaller EpSC niche, which does not give rise to ameloblasts, resulting in a complete lack of enamel deposition on the lingual aspect of the rodent incisor. Thus, enamel, the hardest substance in the body, is secreted uniquely on the labial aspect of the incisor. This leads, to preferential abrasion of the lingual incisor surface during feeding, counteracting the continuous growth of the mouse incisor to produce an incisor of fixed length [10].

Tooth development is regulated by sequential and reciprocal signaling between the epithelium and mesenchyme and is accompanied by patterning and differentiation of specialized cell types at distinct anatomical locations. A complex network of fibroblast (FGFs) and transforming (TGFβ) growth factors regulates proliferation and differentiation of EpSCs during development. The antagonists of these pathways, Sprouty (Spry) proteins and Follistatin (FST), respectively, also regulate EpSC niche development, and growth and asymmetry of the mouse incisor [6], [8].

CTIP2/BCL11B (BCL11B hereafter) is a transcription factor that plays essential roles in the development of the immune [11], [12], central nervous [13], [14], and cutaneous [15] systems and is required for perinatal survival [11]. *Bcl11b^−/−^* incisors and molars are poorly developed, and exhibit a hypoplastic SR. Ameloblasts do not differentiate properly on the labial side, and ectopic ameloblast-like cells form on the lingual side of the *Bcl11b-*null incisor [16].

Our analyses of *Bcl11b^−/−^* mice revealed that BCL11B plays important roles throughout incisor development. Mice lacking *Bcl11b* exhibit epithelial proliferation defects early in development, which ultimately impact incisor size and shape. BCL11B also controls formation of both labial and lingual epithelial stem cell niches and differentiation of ameloblasts. However, BCL11B does so in a bidirectional manner: promoting development and differentiation of the epithelium on the labial side while suppressing that on the lingual side, which strongly enforces asymmetric ameloblast development in the mouse mandibular incisor.

## Materials and Methods

### Mouse Lines

*Bcl11b^−/−^* and *Bcl11b^L2/L2^* mice have been described [15]. Lines carrying mutant alleles of *Spry2* [17] *Spry4* [18], *Fgf3* [19], and *Fgf10* [20], as well as *K14*-cre [21] and *Wnt1*-cre [22] transgenes, were maintained as reported. Animal experiments were approved by the Oregon State University Institutional Animal Care and Use Committee, protocol 4279.

### Histological Analysis, RNA in Situ Hybridization, and Immunohistochemistry

Embryonic heads were fixed in 4% paraformaldehyde, cryopreserved in 30% sucrose, and frozen in O.C.T. Hematoxylin and eosin (H&E) staining and RNA *in situ* hybridization (ISH) with digoxigenin-labeled probes were performed according to standard protocols on 16 µm-thick sagittal sections. Immunohistochemistry using anti-BCL11B (Abcam, 1∶300) was performed as described [23].

### Cell Proliferation Assay

Pregnant mice (E11.5–E16.5) were injected intraperitoneally with 100 µl of 5 mg/ml BrdU solution per 100 g of body weight and sacrificed after 2 h. Cryopreserved heads were serially sectioned (10 µm), and an anti-BrdU antibody (Accurate Chemical, 1∶100) was used to detect BrdU incorporation. The BrdU index was calculated as the mean relative amount of BrdU-positive cells as a fraction of total, DAPI-positive cells. An unpaired, two-tailed Student’s t-test was used to determine statistical significance. At least six sections from a minimum of three animals per genotype and age were analyzed.

### Apoptosis Assay

Apoptosis in sagittal sections (16 µm) was determined with the DeadEnd Colorimetric TUNEL System (Promega) using Cy3-conjugated streptavidin (SA-Cy3; Sigma 1∶250).

## Results

### BCL11B is Expressed at all Stages of Incisor Development

**Figure 1:**
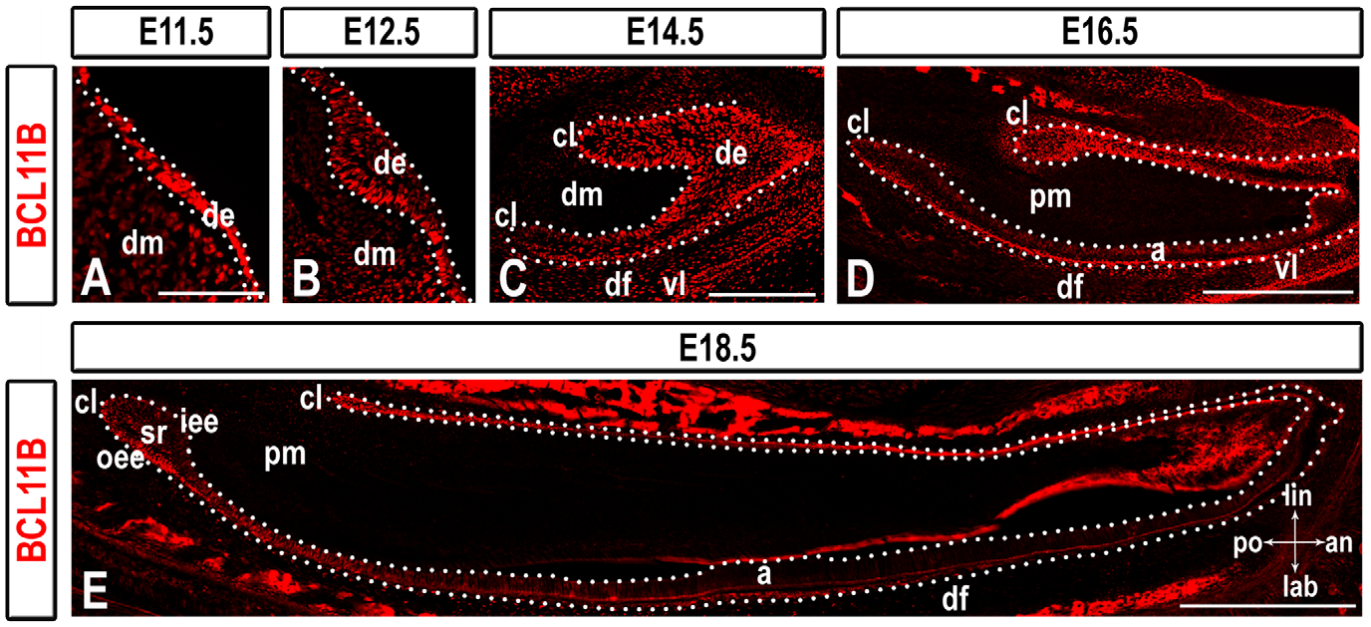
BCL11B expression during incisor development. BCL11B immunostaining in sections of wild-type mice at indicated developmental stages. The epithelium is outlined by white dots. Scale bars: (A-B) 100 µm; (C) 200 µm; (D-E) 500 µm. a, ameloblasts; an, anterior; cl, cervical loop; de, dental epithelium; df, dental follicle; dm, dental mesenchyme; iee, inner enamel epithelium; lab, labial; lin, lingual; oee, outer enamel epithelium; pm, papillary mesenchyme; po, posterior; sr, stellate reticulum; vl, vestibular lamina.

BCL11B is expressed in the ectoderm of the first branchial arch at E9.5 and E10.5 and in the molar at all stages of development [16]. To determine the function of BCL11B in the developing incisor, we analyzed BCL11B expression on sagittal sections of the mandibular incisor from E11.5 to birth. At initiation (E11.5) and early bud (E12.5) stages, BCL11B was expressed in the thickened epithelium; lower levels of BCL11B were detected in the underlying mesenchyme (Figs. 1A and B). High levels of BCL11B persisted in the dental epithelium at cap stage (E14.5), whereas mesenchymal cells surrounding CLs and the follicle continued to express lower levels (Fig. 1C). At early (E16.5) and late (E18.5) bell stages, BCL11B was detected in the lingual epithelium and in the labial OEE, and at lower levels in the papillary mesenchyme surrounding both CLs, dental follicle, SR, and ameloblasts at all stages of differentiation (Figs. 1D, E and S1). BCL11B was expressed in the tissue surrounding the tip of the incisor and the vestibular lamina, an invagination of the oral epithelium that gives rise to the oral vestibule (Figs. 1C and D).

### Reduced Epithelial Proliferation between Initiation and Bud Stage in *Bcl11b* ^−/−^ Incisors

**Figure 2:**
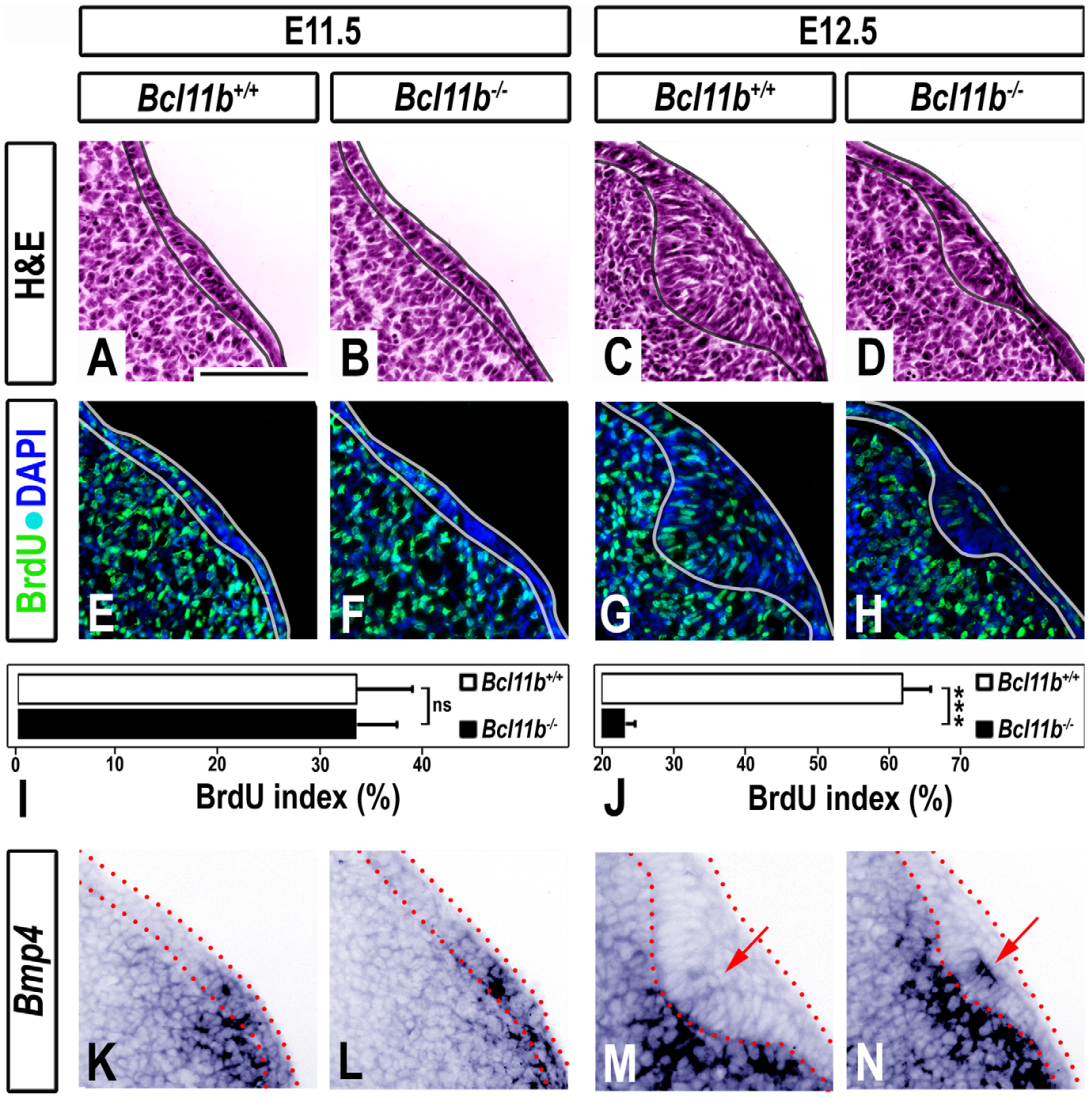
Epithelial invagination defect in *Bcl11b^−/−^* developing incisors between initiation and early bud stage. (A-D) H&E staining in sections of wild-type and *Bcl11b^−/−^* mice at indicated developmental stages. The epithelium is outlined in black. (E-H) BrdU immunostaining (green) in sections of wild-type and *Bcl11b^−/−^* mice. All sections were counterstained with DAPI (blue). The epithelium is outlined in white. (I-J) BrdU index of wild-type and *Bcl11b^−/−^* dental epithelium; *** denotes statistical significance at p ≤ 0.001, n = 3. (K-N) RNA ISH using a *Bmp4* probe in sections of wild-type and *Bcl11b^−/−^* mice at indicated developmental stages. The epithelium is outlined by red dots. Red arrows denote epithelial staining. Scale bar, 100 µm.

The first morphological sign of tooth development is the thickening of the oral epithelium at E11.5. At this stage, wild-type and *Bcl11b* ^−/−^ incisors were morphologically indistinguishable (Figs. 2A, B, E, F, and I; wild-type BrdU index = 33.2±5.4%; *Bcl11b* ^−/−^ BrdU index = 33.2±4.0%). By E12.5 the mutant epithelium appeared approximately one-half the thickness of the wild-type, and cells at the leading edge of the mutant epithelium were less elongated and poorly polarized (Fig. 2C, D). A 2.7-fold decrease in epithelial proliferation of the *Bcl11b* ^−/−^ incisor was detected at E12.5 (Figs. 2G, H, and J; wild-type BrdUindex = 61.9±3.8%; *Bcl11b* ^−/−^ BrdU index =  23.2±1.4%).

Several signaling molecules and transcription factors orchestrate invagination of the epithelium between initiation and early bud stages. For example, BMP4, a critical signaling molecule that regulates tooth initiation and morphogenesis [24], is expressed in the dental epithelium and underlying mesenchyme during the initiation of tooth development at E11.5 (Fig. 2K). *Bmp4* expression largely shifts to the dental mesenchyme by early bud stage in wild-type mice (Fig. 2M) [3], [25]. Alterations in *Bmp4* expression were not detected in *Bcl11b* ^−/−^ incisors at E11.5 (Fig. 2L). However, the *Bcl11b* ^−/−^ epithelium failed to down-regulate expression of *Bmp4* at E12.5 (Fig. 2N). The expression patterns of other critical signaling molecules and transcription factors, including *Activin*, *Shh*, *Pax9*, and *Msx1*, were not altered in *Bcl11b^−/−^* incisors at the bud stage (Fig. S2).

### Altered Development of *Bcl11b^−/−^* Incisors at Cap Stage

**Figure 3:**
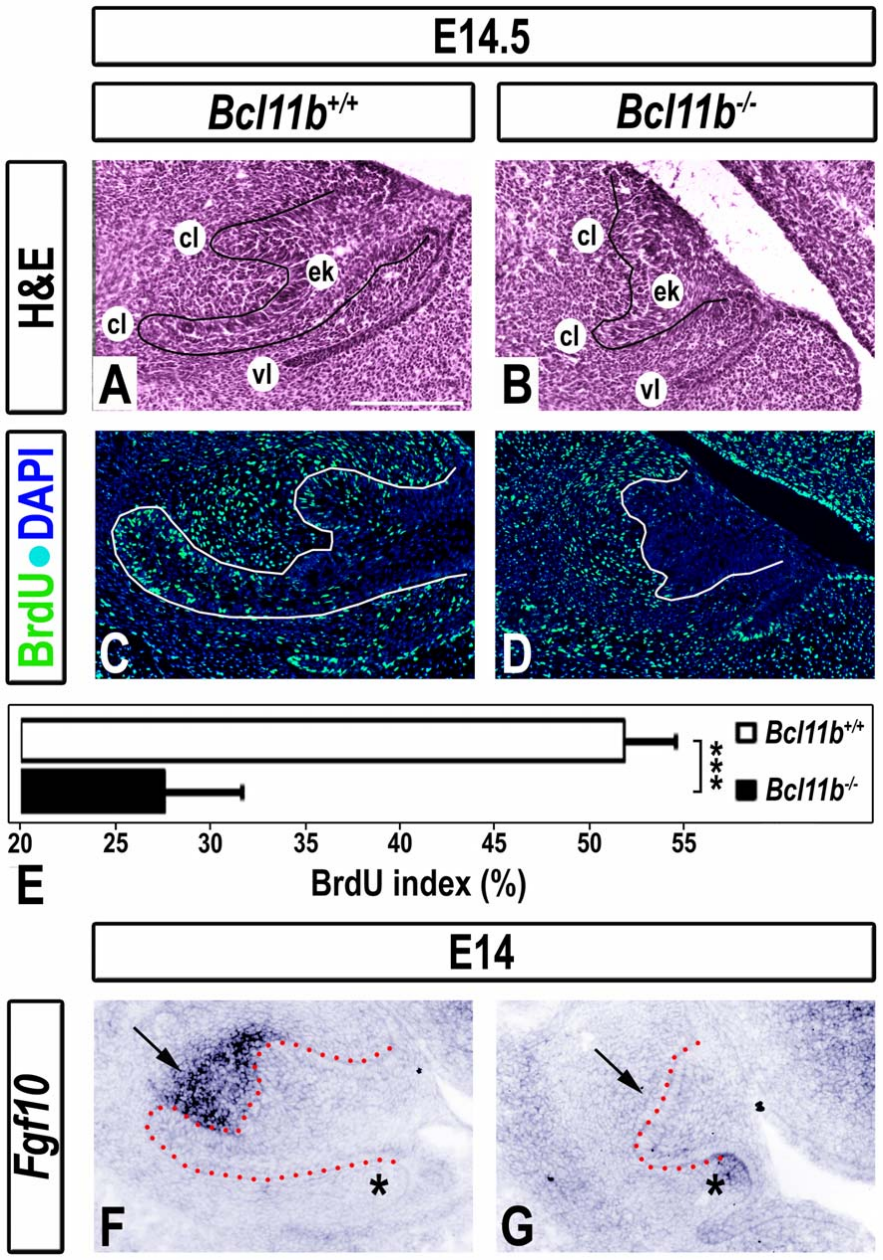
Alterations in *Bcl11b^−/−^* incisor development at cap stage. (A-B) H&E staining in sections of wild-type and *Bcl11b^−/−^* mice at E14.5. The epithelium is outlined in black. (C-D) BrdU immunostaining (green) in sections of wild-type and *Bcl11b^−/−^* mice at E14.5. All sections were counterstained with DAPI (blue). The epithelium is outlined in white. (E) BrdU index of wild-type and *Bcl11b^−/−^* CLs; *** denotes statistical significance at p ≤ 0.001, n = 3. (F-G) RNA ISH using an *Fgf10* probe in sections of wild-type and *Bcl11b^−/−^* mice at E14. Black arrows indicate mesenchymal staining; black asterisks denote the staining between dental epithelium and vestibular lamina. Scale bar, 200 µm. cl, cervical loop; ek, enamel knot; vl, vestibular lamina.

The wild-type, mandibular incisor is characterized by a cap-like shape of the dental epithelium at E14.5, with an enamel knot in the center and protruding CLs (Fig. 3A). The enamel knot is a transitory signaling center that is characterized by minimal proliferation and clearly defined apoptosis [26], [27]. In contrast, the CLs are highly proliferative with a low apoptotic index [28]. The *Bcl11b* ^−/−^ incisor exhibited a delay in epithelial invagination and protrusion of both CLs at E14.5 (Fig. 3B). BrdU-labeling studies revealed that the incisor epithelium of *Bcl11b^−/−^* mice was hypoproliferative compared to that of wild-type mice (27.6±4.1% and 51.8±2.7% BrdU-positive cells, respectively), whereas mesenchymal proliferation appeared unchanged from controls (Fig. 3D and E).

Proliferation of the dental epithelium at cap stage is controlled in part by FGF10, which is derived from mesenchymal cells of the dental papilla [29]. The *Bcl11b* ^−/−^ papillary mesenchyme was essentially devoid of *Fgf10* transcripts at early E14 and ectopic *Fgf10* expression was noted between the dental epithelium and vestibular lamina, the latter of which exhibited impaired invagination (Fig. 3F, G). The delay of initiation of mesenchymal *Fgf10* expression may contribute to decreased dental epithelial proliferation, delayed invagination of the dental epithelium, and subsequently decreased size of the mutant incisor.

The expression patterns of *Shh*, *Gli1*, *Fgf3*, *Fgf9*, *Spry2*, *Spry4*, *Bmp4*, *activin*, *Fst*, and *Tbx1*, were unaltered in *Bcl11b^−/−^* incisors at cap stage (Fig. S3).

Apoptotic cells were predominantly localized in the enamel knot of wild-type incisors at E14.5 (Fig. S4A). However, very few apoptotic cells were detected in the *Bcl11b* ^−/−^ enamel knot (Fig. S4B), consistent with delayed incisor development in *Bcl11b* ^−/−^ mice.

### Reduced Size and Disruption of Labial-lingual Asymmetry at Bell Stage in *Bcl11b* ^−/−^ Incisors

**Figure 4:**
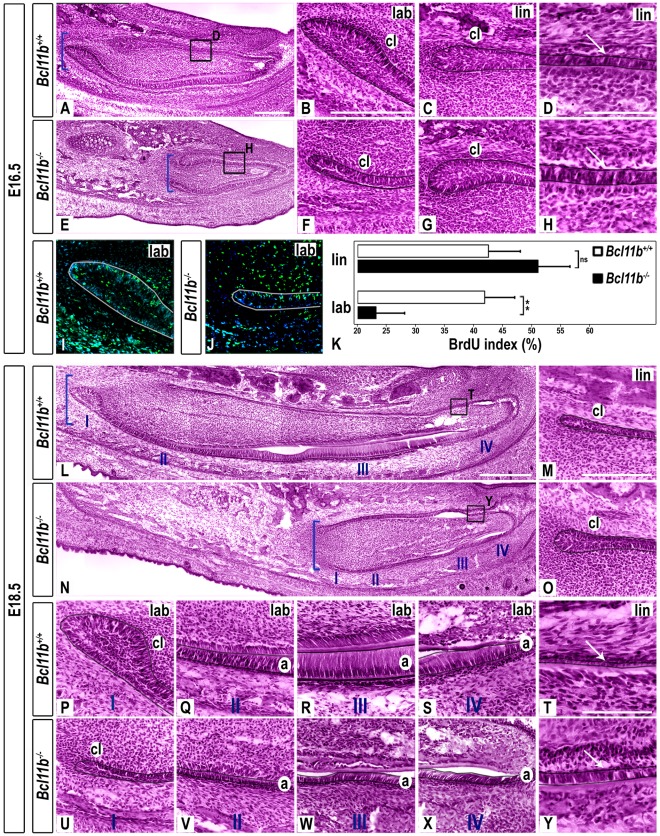
Morphological defects in *Bcl11b^−/−^* incisor development at bell stage. (A-H, L-Y) H&E staining in sections of wild-type and *Bcl11b^−/−^* mice at indicated stages. The epithelium is outlined in black. Brackets indicate the posterior end of the incisors in A, E, L, N. Panels D, H, T, Y are higher magnification images of boxes in A, E, L, N, respectively. White arrows indicate lingual epithelium. I denotes the labial CL; II-IV denote stages of progression of ameloblast differentiation in L, N, P-S, U-X. The *Bcl11b^−/−^* labial CL was smaller than that of wild-type mice by 60% at E16.5 and by 69% at E18.5 (p ≤ 0.001, n = 11), whereas the mutant lingual CL was enlarged by 35% at E16.5 and by 64% at E18.5 relative to wild-type tissue (p ≤ 0.001, n = 11). (I-J) BrdU immunostaining (green) in sections of wild-type and *Bcl11b^−/−^* mice at E16.5. All sections were counterstained with DAPI (blue). The epithelium is outlined in white. (K) BrdU index of wild-type and *Bcl11b^−/−^* basal epithelium of labial and lingual CLs; ns, not significant; *** denotes statistical significance at p ≤ 0.001, n = 3. Scale bars: (A-S, U-X) 200 µm; other panels, 100 µm. a, ameloblasts; cl, cervical loop; lab, labial; lin, lingual.

Wild-type incisors at E16.5 are characterized by a large labial CL, which contains stem cells that give rise to ameloblasts (Figs. 4A and B). In contrast, the lingual CL of wild-type incisors is relatively smaller, consistent with reduced developmental potential on the lingual side of the incisor (Fig. 4C). *Bcl11b* ^−/−^ incisors were reduced in size by approximately half at this stage (compare Figs. 4A and E) and were characterized by a hypocellular labial CL (compare Figs. 4B and F), an enlarged lingual CL (compare Figs. 4C and G), and elongated cells resembling ameloblasts along the length of the lingual epithelium (compare Figs. 4D and H).

The posterior basal epithelium of the labial CL of *Bcl11b^−/−^* mice was hypoproliferative relative to that of wild-type mice at E16.5 (23.2±4.9% and 42.0±5.0% BrdU-positive cells, respectively; Figs. 4I-K). However, neither the apoptotic index (Figs. S4C and D) nor proliferation in the lingual CL (Fig. 4K) of mutant incisors was significantly different from wild-type.

By E18.5, wild-type incisors developed a large labial (region I, Figs. 4L and P) and a small lingual (Figs. 4L and M) CL. Ameloblast differentiation occurs sequentially in the labial IEE with mitotic pre-ameloblasts (Figs. 4L and Q), post-mitotic secretory ameloblasts (Figs. 4L and R), and mature ameloblasts (Figs. 4L and S) in regions II, III, and IV, respectively [9]. Ameloblasts are not present on the lingual side of the wild-type incisor, but rather a thin layer of non-polarized epithelial cells is found on this aspect of the developing tooth (Figs. 4L and T).

*Bcl11b^−/−^* incisors were reduced in size at E18.5 (compare Figs. 4L and N). However, the mutant lingual CL was enlarged (compare Figs. 4M and O), and the labial CL was markedly hypoplastic (compare Figs. 4P and U) to the point of resembling the lingual CL of wild-type mice in both size and morphology (compare Figs. 4M and U). Mutant ameloblasts were smaller and disorganized at all stages of differentiation along the labial epithelium (compare Figs. 4Q-S and V-X), and an abnormal layer of polarized cells resembling ameloblasts was observed in the anterior region of the lingual epithelium (compare Figs. 4T and Y). Finally, *Bcl11b* ^−/−^ incisors were approximately one-half the length of wild-type incisors at birth and correspondingly narrower across the entire tooth (Fig. S5).

These results demonstrate that BCL11B plays an important role in development of the labial CL and differentiation of ameloblasts, while simultaneously suppressing these processes on the lingual side of the incisor.

### Delay in Ameloblast Development and Ectopic Formation of Lingual Ameloblast-like Cells in *Bcl11b^−/−^* Incisors

**Figure 5:**
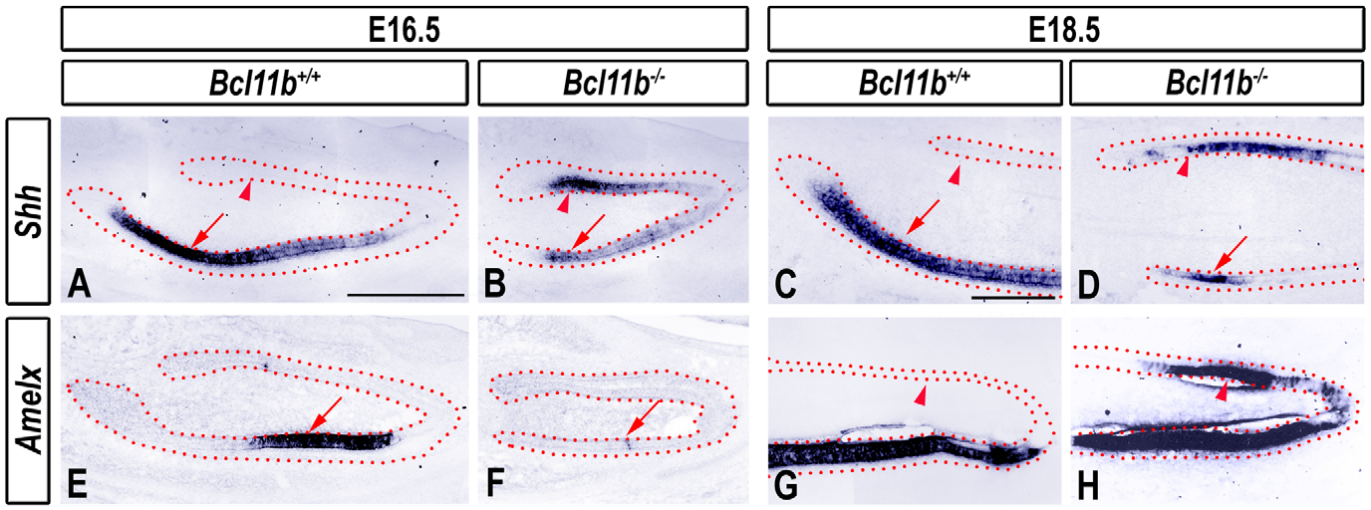
Altered ameloblast development in *Bcl11b^−/−^* incisors. RNA ISH using the indicated probes in sections of wild-type and *Bcl11b^−/−^* mice at indicated developmental stages. The epithelium is outlined by red dots. Red arrows and arrowheads denote labial and lingual epithelial staining, respectively. Scale bars: (A-B, E-F) 500 µm; other panels, 200 µm.

To determine if labial ameloblasts and lingual ameloblast-like cells underwent differentiation in *Bcl11b^−/−^* incisors, we examined expression of sonic hedgehog (*Shh*) and amelogenin (*Amelx*), markers of pre-ameloblasts [6], [30] and mature ameloblasts [31], respectively. *Shh* expression was observed in a gradient along the length of the labial IEE of wild-type incisors at E16.5 and E18.5, with the most intense staining in the posterior region (Fig. 5A, C). *Shh* expression was greatly reduced in the labial epithelium of *Bcl11b^−/−^* mice, and ectopic *Shh* transcripts were detected in the lingual epithelium at E16.5 and E18.5 (Figs. 5B and D). The expression pattern of *Gli1*, a mediator of SHH signaling [32], reflected changes in *Shh* expression in the mutant incisor (Fig. S6).

*Amelx* expression was greatly reduced in the labial epithelium of *Bcl11b^−/−^* mice at E16.5 (compare Figs. 5E and F) but recovered to a level similar to that of wild-type mice by E18.5 (Fig. 5G). Ectopic *Amelx* expression was observed in the anterior lingual IEE of *Bcl11b^−/−^* mice at E18.5 (compare Figs. 5G and H), consistent with the presence of terminally differentiated ameloblasts in the lingual epithelium.

These results demonstrate that BCL11B plays a key role in the establishment and/or enforcement of developmental incisor asymmetry and cellular differentiation within the ameloblast lineage.

### Alteration of the FGF Signaling at Bell Stage in *Bcl11b^−/−^* Incisors

**Figure 6:**
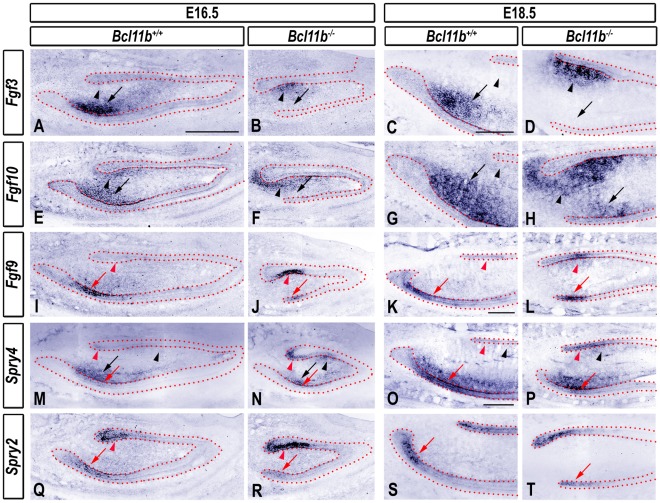
Labial to lingual reversal of expression of FGF and Sprouty genes in *Bcl11b^−/−^* incisor. RNA ISH using the indicated probes in sections of wild-type and *Bcl11b^−/−^* incisors at indicated developmental stages. The epithelium is outlined by red dots. Black and red arrows denote labial mesenchymal and epithelial staining, respectively, and black and red arrowheads indicate lingual mesenchymal and epithelial staining, respectively. Scale bars: (A-B, E-F, I-J, M-N, Q-R) 500 µm; other panels, 200 µm.

Asymmetric development of the CLs is controlled by several signaling pathways. FGFs and their intracellular antagonists, the Sprouty proteins, are crucial for proper development of the labial and lingual CLs [6]. FGF3 and FGF10 are key mesenchymal instructive signals that cooperatively stimulate proliferation of the incisor epithelium at bell stage [5], [33], [34]. *Fgf3* is expressed exclusively within the posterior labial mesenchyme in wild-type incisors (Figs. 6A and C), whereas *Fgf10* transcripts are more widely distributed around the labial CL and to a lesser extent in the lingual mesenchyme (Figs. 6E and G).

The *Fgf3* expression domain, which is located in the mesenchyme just anterior to the labial CL in wild-type mice, was absent in *Bcl11b* ^−/−^ mutants at E16.5 and E18.5. However, *Fgf3* was ectopically expressed in the mesenchyme adjacent to the lingual CL in *Bcl11b* ^−/−^ mice (Figs. 6B and D). The expression pattern of *Fgf10* was altered in *Bcl11b* ^−/−^ incisors in a manner that was qualitatively similar to that of *Fgf3* (Figs. 6F and H).

Epithelial FGF9 forms a positive-feedback signaling loop with mesenchymal FGF3 and FGF10 on the labial side of the wild-type incisor [6]. *Fgf9* RNA was detected anterior to the labial CL of the wild-type incisor (Figs. 6I and K). This *Fgf9*-positive domain was reduced in *Bcl11b* ^−/−^ incisors, and ectopic expression of *Fgf9* was detected in the lingual epithelium at E16.5 and E18.5 (Figs. 6J and L).

Sprouty proteins are responsible, in part, for inhibition of ameloblast differentiation in the lingual epithelium [6]. *Spry4* RNA was detected in the mesenchyme adjacent to the labial CL, and at lower levels in the posterior lingual and labial epithelium in wild-type mice at E16.5 and E18.5 (Figs. 6M and O). *Spry2* expression was detected predominantly in the posterior lingual and labial epithelium of the wild-type incisor (Figs. 6Q and S).

*Spry4* expression was up-regulated in the lingual basal epithelium and underlying mesenchyme of *Bcl11b^−/−^* incisors at E16.5 and E18.5, and down-regulated on the labial side of the developing *Bcl11b^−/−^* incisor at both developmental stages (Figs. 6N and P). *Spry2* expression was up-regulated in the lingual CL and slightly down-regulated in the labial epithelium of *Bcl11b^−/−^* mice at E16.5 and E18.5 (Figs. 6R and T).

Mesenchymal FGF10 stimulates expression of Lunatic Fringe (*Lfrn*), which encodes a secretory molecule that modulates the Notch pathway [5]. To determine if the Notch pathway was altered in *Bcl11b^−/−^* incisors at bell stage, we examined expression patterns of *Lfrn* and *Notch1*. *Lfrn* RNA was detected predominantly along the length of IEE and in the posterior OEE of wild-type incisors at E16.5 and E18.5 (Figs. S7A and C). *Lfrn* expression was down-regulated at the posterior end of the labial CL of *Bcl11b^−/−^* incisors at both E16.5 and E18.5 (Figs. S7B and D). Ectopic *Lfrn* expression was detected in the posterior part of the mutant lingual epithelium at E18.5 (Fig. S7D). Loss of BCL11B did not affect the level of expression or localization of *Notch1* transcripts. However, *Notch1* expression reflected the morphological expansion and contraction of lingual and labial SR, respectively, in *Bcl11b^−/−^* incisors (Figs. S7E-H).

These findings highlight dysregulation of the FGF signaling pathways as being central to the incisor phenotype of *Bcl11b^−/−^* mice. As asymmetric expression of *Fgf3* and *Fgf10* contribute to asymmetric development of labial and lingual EpSC niches [28]. Thus, the complete reversal of asymmetric *Fgf3* and *Fgf10* expression, together with that of *Fgf9*, likely underlies the enhanced and repressed development of the lingual and labial CLs, respectively, in *Bcl11b^−/−^* mice.

### Disruption of TGFβ Signaling at Bell Stage in *Bcl11b^−/−^* Mice

The TGFβ family members, BMP4 and activin βA, and the antagonist FST play key roles in the generation and maintenance of asymmetric ameloblast localization during incisor development. For example, FST inhibits ameloblast differentiation on the lingual side of the incisor, whereas BMP4 promotes it on the labial side. In contrast, activin enhances development of the labial CL, whereas BMP4 limits CL growth [24], [28], [35].

**Figure 7:**
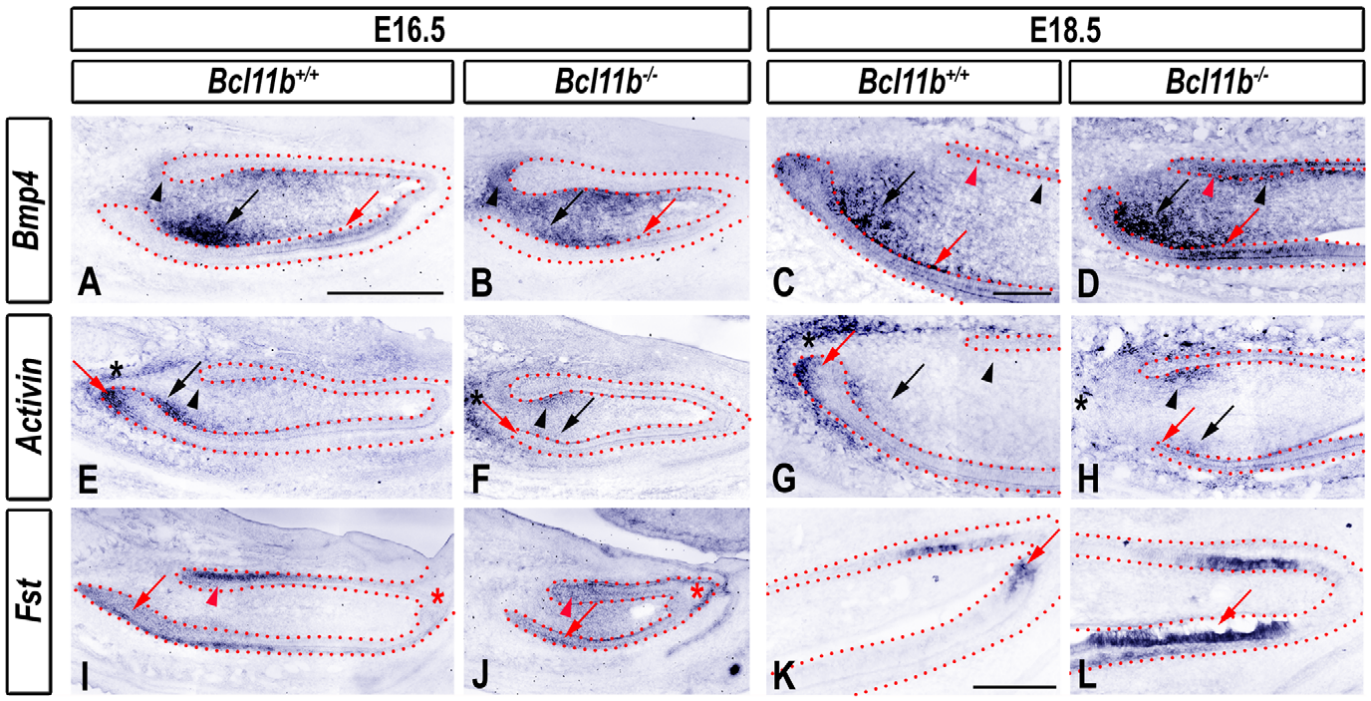
Altered expression of TGFβ genes and *Fst* in *Bcl11b^−/−^* incisor. RNA ISH using the indicated probes in sections of wild-type and *Bcl11b^−/−^* incisors at indicated developmental stages. The epithelium is outlined by red dots. Black and red arrows denote labial mesenchymal and epithelial staining, respectively, and black and red arrowheads indicate lingual mesenchymal and epithelial staining, respectively. Black and red asterisks denote staining in the posterior part of the dental follicle and epithelial tip of the incisor, respectively. Scale bars: (A-B, E-F, I-J) 500 µm; other panels, 200 µm.

*Bmp4* expression was detected predominantly in the labial mesenchyme, anterior to the labial CL, in wild-type mice at E16.5. Lower levels of *Bmp4* transcripts were present in the mesenchyme underlying the lingual epithelium and in an anterior region of the ameloblast layer (Fig. 7A). The boundaries of mesenchymal *Bmp4* expression were disrupted in *Bcl11b* ^−/−^ incisors at E16.5, with ectopic expression noted in the mesenchyme posterior to the lingual CL. Expression of *Bmp4* in the ameloblast layer appeared reduced in mutants at this stage (Fig. 7B). At E18.5, *Bmp4* transcripts were detected predominantly in the labial epithelium, in a wide region of labial mesenchyme, and at lower levels on the lingual side of the wild-type incisor (Fig. 7C). *Bmp4* expression increased uniformly in all of these domains in *Bcl11b* ^−/−^ mutants at E18.5 (Fig. 7D).

*Activin* expression was restricted to the labial mesenchyme directly underlying the posterior epithelium, within the tip of the labial CL, and in the posterior part of the dental follicle in wild-type mice at E16.5 and E18.5 (Figs. 7E and G). *Activin* expression was lost within the labial mesenchyme and epithelium in *Bcl11b* ^−/−^ mice at both developmental stages. However, ectopic mesenchymal expression of *activin* was observed around the lingual CL and follicular expression of *activin* appeared to be delocalized in the *Bcl11b* ^−/−^ incisors at E16.5 and E18.5 (asterisks in Figs. 7F and H).

*Fst* transcripts were observed in the OEE on the labial and lingual sides at E16.5 (Fig. 7I; see also [35]). *Fst* expression in the OEE persisted at E18.5, and *Fst* transcripts were also detected in highly-defined domains at the anterior epithelial tip of the incisor on both labial and lingual sides (Fig. 7K; data not shown). In contrast, *Fst* transcripts were diffusely distributed throughout the labial and lingual epithelium of *Bcl11b^−/−^* incisors, particularly at the anterior (incisal) tip of the epithelium, and in the papillary mesenchyme at E16.5 (Fig. 7J). *Fst* expression within the posterior region of the wild-type incisor at E18.5 was indistinguishable from that of *Bcl11b* ^−/−^ mice (data not shown). However, we noted a dramatic expansion of the *Fst* expression domain within the anterior labial epithelium at E18.5. Additionally, *Bcl11b* ^−/−^ incisors failed to extinguish *Fst* expression along the length of the labial OEE at E18.5 (Fig. 7L).

### Cell Autonomous Effects of BCL11B in Lingual Epithelium

**Figure 8:**
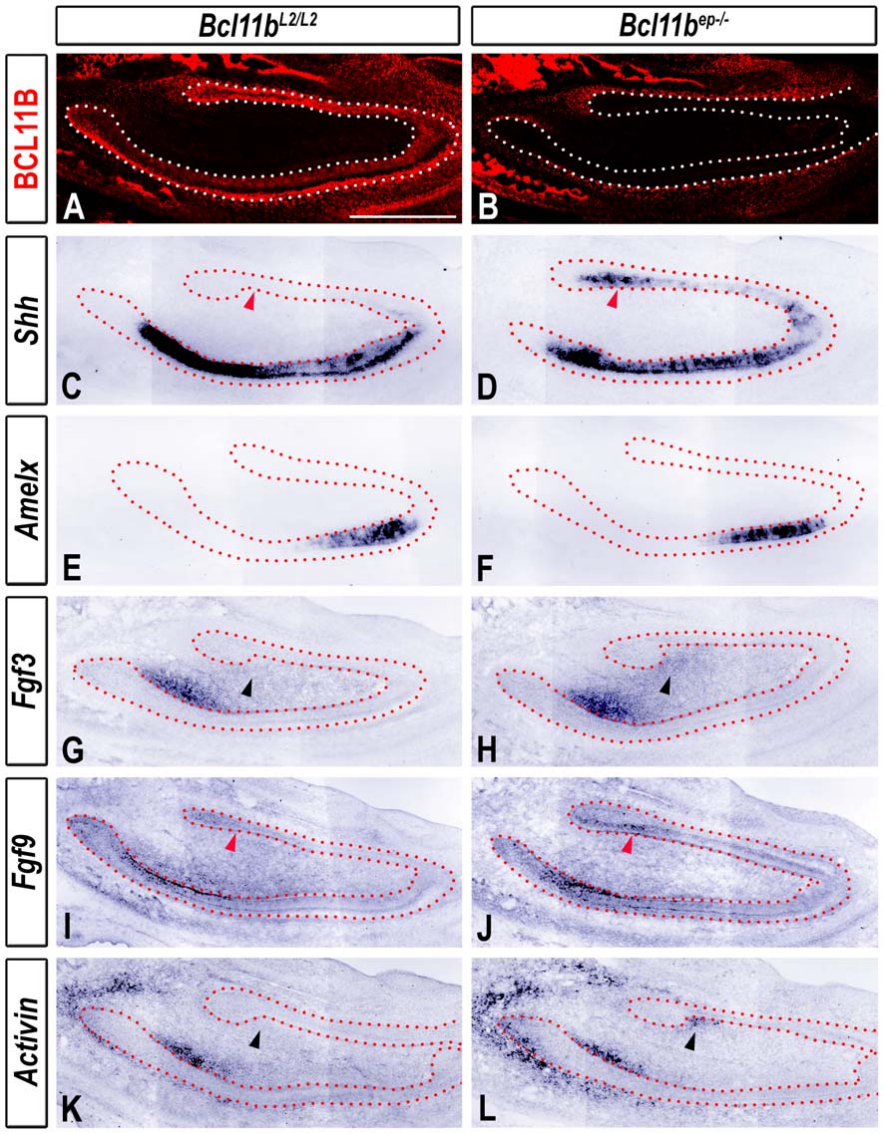
Ectopic lingual expression of ameloblast markers and signaling molecules in *Bcl11b^ep−/−^* incisor. (A-B) BCL11B immunostaining in sections of *Bcl11b^L2/L2^* and *Bcl11b^ep−/−^* mice at E16.5. The epithelium is outlined by white dots. (C-L) RNA ISH using the indicated probes in sections of *Bcl11b^L2/L2^* and *Bcl11b^ep−/−^* incisors at E16.5. The epithelium is outlined by red dots. Black and red arrowheads denote lingual mesenchymal and epithelial staining, respectively. Scale bar, 500 µm.

BCL11B is expressed in both ectodermal-derived epithelium and neural crest-derived mesenchyme (Fig. 1). We created lines conditionally null for *Bcl11b* expression in both germinal layers to determine the expression domain responsible for BCL11B-mediated suppression of ameloblast differentiation in the lingual epithelium. Mice harboring an epithelial-specific deletion of *Bcl11b* (*Bcl11b^ep−/−^*), which were created by crossing floxed *Bcl11b^L2/L2^* mice with the *K14-*cre deleter strain [21], clearly lacked BCL11B in the entire dental epithelium (Figs. 8A and B). However, specific BCL11B expression persisted in the dental mesenchyme and other non-epithelium-derived tissues. *Bcl11b^ep−/−^* mice expressed the pre-ameloblast marker *Shh* with an ectopic gradient along the length of the lingual epithelium at E16.5 (Figs. 8C and D), and this persisted at a lower level at E18.5 (Fig. S8B) However, *Bcl11b^ep−/−^* mice did not express *Amelx* in the lingual epithelium at either E16.5 (Fig. 8F) or E18.5 (Fig. S8E). Considered together, these results suggest that *Bcl11b^ep−/−^* mice initiate but do not complete ameloblast differentiation within the lingual dental epithelium.

Next, we examined the expression of several genes encoding signaling molecules to determine the effect of epithelium-specific inactivation of *Bcl11b* on generation of labial-lingual asymmetry at E16.5. A low level of ectopic expression of *Fgf3*, *Fgf9*, and *activin* was detected on the lingual side of the *Bcl11b^ep−/−^* incisor, and this was qualitatively similar to *Bcl11b^−/−^* incisors (compare Figs. 8G-L, Fig. 6B and F, and Fig. 7F). However, we did not observe alterations in the expression patterns of these signaling molecules on the labial side of the *Bcl11b^ep−/−^* incisor (Figs. 8G-L), as described previously for *Bcl11b^−/−^* mice (see Figs. 6 and 7).

The size and shape of the *Bcl11b^ep−/−^* incisors were similar to control incisors (see Fig. 8), and the slight variations in lingual gene expression in *Bcl11b^ep−/−^* incisors did not result in altered amelogenesis as determined by X-ray micro-CT radiography performed on P21 mandibles (Fig. S9).

Excision of the *Bcl11b* locus in neural crest-derived mesenchyme using the *Wnt1*-cre deleter line (*Bcl11b^mes−/−^*; see Figs. S10A and B) did not result in altered morphology or disrupted gene expression patterns (*Shh*, *Amelx*, *Fgf3*, and *activin*; Figs. S10C-J; see also S8C and F).

These data indicate that epithelial, but not mesenchymal *Bcl11b* expression is required for suppression of ectopic pre-ameloblast formation in the lingual epithelium. However, loss of *Bcl11b* in the epithelium is not sufficient for the lingual pre-ameloblasts to persist or to undergo further differentiation into mature, *Amelx-*positive ameloblasts.

### FGF Signaling Negatively Regulates BCL11B Expression in the Lingual IEE and SR

The ectopic development of lingual pre-ameloblasts expressing *Shh* in *Bcl11b^−/−^* mice is similar to that reported in *Spry4^−/−^; Spry2^+/−^* mice. Loss of Sprouty gene expression results in abnormal FGF gene expression and establishment of a FGF positive-feedback signaling loop on the lingual side of the incisor. In addition, *Spry4^−/−^; Spry2^+/−^* mice were characterized by up-regulated expression of *Etv4* and *Etv5* (previously known as *Pea3* and *Erm*), which are considered to be transcriptional targets of FGF signaling, and indicative of activation of the FGF signaling pathway(s) in mutant incisors [6], [36], [37]. We assessed expression of BCL11B in incisors from *Spry4^−/−^; Spry2^+/−^* embryos in order to determine if the FGF signaling pathway(s) regulates BCL11B expression.

**Figure 9:**
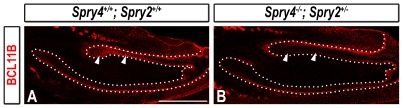
Inhibition of *Bcl11b* expression in the lingual IEE of *Spry4^−/−^; Spry2^+/−^* mice at E16.5. BCL11B immunostaining in sections of wild-type and *Spry4^−/−^; Spry2^+/−^* mice at E16.5. The epithelium is outlined by white dots. White arrowheads denote lingual epithelial staining. Scale bar, 500 µm.

BCL11B was highly expressed in the entirety of the wild-type lingual epithelium at E16.5, including the CL, anterior OEE, and IEE (Fig. 9A; see also Fig. 1D). In contrast, BCL11B protein levels were dramatically decreased in the *Spry4^−/−^; Spry2^+/−^* incisor, particularly within the lingual IEE and SR, while BCL11B levels in the lingual OEE and labial epithelium were largely unaffected (Fig. 9B).

Expression of *Tbx1*, which is also important for incisor developmental asymmetry, was increased in the lingual epithelium of *Spry4^−/−^; Spry2^+/−^* incisors [38]. These findings prompted us to examine *Tbx1* expression in *Bcl11b^−/−^* mice. *Tbx1* was predominantly expressed in the posterior basal epithelium on the labial side of wild-type incisors at both E16.5 and E18.5, and diffusely at a much lower level in the lingual epithelium (Figs. S11A and C). We observed striking up-regulation of *Tbx1* expression in the lingual IEE of *Bcl11b* ^−/−^ mice at E16.5 and E18.5 (Figs. S11B and D), suggesting that BCL11B directly or indirectly represses the *Tbx1* expression in the lingual epithelium, and that up-regulation of *Tbx1* expression in *Spry4^−/−^; Spry2^+/−^* mice [38] may occur through down-regulation of BCL11B protein levels. These findings place BCL11B downstream of FGF signaling and upstream of *Tbx1* expression in the lingual epithelium of the developing incisor. *Tbx1* expression was severely decreased in the labial epithelium of *Bcl11b^−/−^* mice at E16.5 (Fig. S11B). Labial expression of *Tbx1* in the *Bcl11b^−/−^* incisor recovered by E18.5 (Fig. S11D), suggesting that another factor(s) may compensate for loss of BCL11B expression in the control of expression of *Tbx1* in the labial epithelium.

These above findings suggest that the FGF signaling pathways regulate BCL11B expression in the lingual epithelium, and we hypothesized that inactivation of FGF signaling may lead to up-regulation of BCL11B expression within the labial IEE. In order to test this hypothesis, we assessed BCL11B expression in *Fgf3^−/−^; Fgf10^+/−^* incisors; however, BCL11B immunostaining was indistinguishable from wild-type incisors (Fig. S12). It is conceivable that another FGF family member(s) may compensate for loss of *Fgf3* expression and partial loss of *Fgf10* expression by enforcing the repression of BCL11B expression within the labial IEE [39]. Indeed, *Fgf3^−/−^; Fgf10^+/−^* and wild-type incisors are nearly identical in size (Fig. S12), suggesting that loss or partial loss of these two signaling molecules did not compromise proliferation during incisor development. Finally, it is possible that regulation of BCL11B expression within the labial epithelium may not involve the FGF signaling pathways, as was clearly evident on the lingual side (Fig. 9B).

## Discussion

The studies reported here demonstrate that the transcription factor BCL11B participates in several essential aspects of mouse incisor development. First, BCL11B controls epithelial proliferation, which ultimately impacts the size and shape of the incisor. Second, BCL11B plays a key role in the establishment and maintenance of labial-lingual asymmetry by regulating the expression of several key signaling molecules and transcription factors. Third, BCL11B is essential for the proper formation, differentiation, and localization of ameloblasts.

To our knowledge, this is the first report of a transcription factor that integrates developmental control of both labial and lingual EpSC niches and ameloblasts. Such regulation appears to be bidirectional: BCL11B stimulates the development of the labial CL by enhancing the expression of key signaling molecules on the labial side while limiting development of the lingual CL by repressing the expression of the same signaling molecules on the lingual aspect. Subsequently, BCL11B promotes differentiation of the labial, EpSC-derived IEE cells into mature ameloblasts and blocks ectopic formation and differentiation of the lingual IEE into cells of the ameloblast lineage.

### BCL11B Regulates Proliferation of the Dental Epithelium

BCL11B is initially required for proper transition from initiation to early bud stage of tooth development. Specifically, BCL11B is necessary for the proper timing of epithelial proliferation, invagination, and down-regulation of epithelial *Bmp4* expression. During tooth initiation, BMP4 is secreted by the epithelium and induces the mesenchymal expression of genes (*Msx1*, *Msx2*, and *Bmp4*) that further direct incisor formation [4]. While the significance of down-regulation of *Bmp4* expression in the dental epithelium at bud stage is unknown, overexpression of *Bmp4* in the distal respiratory epithelium results in decreased epithelial proliferation [40]. Thus, a delay in down-regulation of epithelial *Bmp4* expression may contribute to reduced proliferation of the dental epithelium between initiation and bud stages.

The size and shape of the incisor is tightly regulated by the opposing forces of cellular proliferation and apoptosis, both of which are controlled by signaling pathways. FGF10 induces a mitogenic response in dental epithelial cells [29], and a delay in induction of *Fgf10* in *Bcl11b^−/−^* incisors may further contribute to the proliferation defect in *Bcl11b^−/−^* dental epithelium. Therefore, a combination of at least two molecular dysregulations, a delay in down-regulation of epithelial *Bmp4* expression at E12.5 and in the induction of mesenchymal *Fgf10* expression at E14, may contribute to decreased proliferation of *Bcl11b^−/−^* dental epithelium. We further propose that altered epithelial proliferation in the absence of BCL11B may account for the slowed invagination of this tissue, resulting in an incisor that is approximately one-half of the size of a wild-type incisor at birth.

Apoptosis of epithelial cells comprising the enamel knot at cap stage also regulates the overall shape of the incisor [27]. Thus, delayed initiation of apoptosis in *Bcl11b^−/−^* incisors may also contribute to altered morphology in the mutant.

### BCL11B Controls the Expression of FGF and TGFβ Family Members

Transition from the cap to bell stage of incisor development is accompanied by establishment of an asymmetric shape. The size and asymmetry of the mouse incisor is dictated by transcription factor networks, which control the expression of genes encoding components of various signaling pathways that play deterministic roles in cellular specification and organogenesis. The FGF and TGFβ signaling pathways, and their respective antagonists–the Sprouty proteins and FST–are particularly important in incisor development.

FGF3 and FGF10, both of which are expressed predominantly in the labial mesenchyme, maintain proliferation of EpSCs and, thus, directly contribute to the asymmetric shape of the incisor [5], [28], [33]. Furthermore, both FGF3 and FGF10, together with epithelial FGF9, form a positive-feedback loop on the labial aspect of the tooth. This FGF feedback loop is inhibited by Sprouty proteins on the lingual side, resulting in the limited development of the lingual EpSC niche [6]. In turn, the expression of the Sprouty genes can be induced by FGF signaling [41].

In the absence of BCL11B, a remarkable inversion of the expression patterns of FGF genes relative to the labial-lingual axis occurred, such that these genes were expressed predominantly on the lingual side, with no or little expression was observed on the labial side. Therefore, BCL11B may function as a spatial switch governing expression of FGF signaling pathway members (Fig. S13). Expression of Sprouty genes was altered in a similar manner in *Bcl11b^−/−^* incisors, possibly in a compensatory or feedback manner. Consistent with this, expression of *Tbx1*, which is positively regulated by FGF signaling in the developing incisor [38], was similarly altered. Our findings strongly suggest that complete loss of expression of FGF family members on the labial side coupled with ectopic expression of these signaling proteins on the lingual side of the *Bcl11b* ^−/−^ incisor underlies the abnormal morphology of the *Bcl11b^−/−^* tooth.

The FGF and TGFβ signaling pathways are closely interweaved during incisor development. For example, BMP4 represses *Fgf3* expression in the mesenchyme; however, activin abrogates this repression on the labial side of the incisor, which allows FGF3 expression within this domain. Low activin expression in the lingual mesenchyme allows BMP4 to inhibit *Fgf3* expression on the lingual side in an unopposed fashion [28]. In addition, BMP4 promotes ameloblast differentiation within the labial epithelium, possibly by inducing expression of *p21* and ameloblastin. Ameloblast-inducing activity of BMP4 is inhibited on the lingual side by FST, but the relative lack of *Fst* expression in the labial epithelium facilitates terminal differentiation of ameloblasts [35]. Alteration of *Bmp4* expression *in Bcl11b^−/−^* incisor generally paralleled those observed with FGF family members. Thus, BCL11B appears to be required for *Bmp4* expression on the labial side of the developing incisor and for suppression of *Bmp4* expression in the lingual mesenchyme.

Expression of *activin* also exhibited complete reversal in the mutants at the bell stage. *Activin* expression was completely lost on the labial side of the mutant incisor, perhaps allowing BMP4 to inhibit *Fgf3* expression within this domain. In contrast, *activin* transcripts were detected in the lingual mesenchyme of *Bcl11b* ^−/−^ incisors, suggesting that this ectopic expression domain allows activin to block the repressive action of BMP4 on *Fgf3* expression in this tissue. Because FGF3 functions in a positive-feedback loop with FGF10 and FGF9 [6], the expression patterns of the latter were also altered. FGF3, and possibly FGF family members, could then induce the development of the lingual EpSC niche in *Bcl11b* ^−/−^ mice. Thus, we propose that activin contributes to the establishment of new borders of expression of FGF family members in the *Bcl11b* ^−/−^ incisor.

### BCL11B Controls Asymmetric Development of the Ameloblasts

 The asymmetric pattern of expression of FGF and TGFβ signaling molecules is thought to lead to the asymmetric development of ameloblasts in wild-type incisors. Therefore, dysregulated expression of these signaling pathways in *Bcl11b^−/−^* incisors likely contributes to the ectopic development of lingual, ameloblast-like cells and delayed development of the labial ameloblasts. The expanded lingual EpSC niche in *Bcl11b^−/−^* incisors gave rise to ectopic *Shh*-expressing pre-ameloblasts, which further differentiated into mature, *Amelx*-positive ameloblasts. The mutant labial EpSC niche also gave rise to some pre-ameloblasts, which were characterized by down-regulated *Shh* expression. These pre-ameloblasts failed to differentiate into mature ameloblasts at the early bell stage. Although the pool of *Shh*-positive pre-ameloblasts was greatly reduced in *Bcl11b^−/−^* incisors at late bell stage, it was remarkable that differentiation to *Amelx*-positive ameloblasts occurred in the labial epithelium by E18.5. This observation suggests that another transcription factor(s) may compensate for loss of *Bcl11b* expression in the ameloblast lineage, allowing ameloblast development to occur, albeit in a delayed manner.

Ectopic *Shh*-positive pre-ameloblasts were abundant on the lingual aspect of the *Bcl11b^ep−/−^* incisor at early bell stage. Low levels of ectopic lingual expression of *Fgf3*, *Fgf9*, and *activin* might contribute to such differentiation of IEE. However, the ectopic, lingual *Shh*-positive domain was dramatically reduced in size and was present only in the posterior epithelium by late bell stage, and mature ameloblast-like cells were not observed on the lingual aspect of the *Bcl11b^ep−/−^* incisor, suggesting that other factors and/or mesenchymal BCL11B may be sufficient to suppress terminal differentiation in the ameloblast lineage within the lingual epithelium. Deletion of *Bcl11b* in either the epithelium or mesenchyme did not affect labial expression of ameloblast markers and key signaling molecules, the morphology of the labial CL, ameloblast development or amelogenesis, suggesting that both epithelial and mesenchymal BCL11B may contribute to developmental processes on the labial side of the incisor.BCL11B regulates expression of signaling molecules and transcription factors that are essential for establishment and maintenance of asymmetric incisor development. The majority of these changes in expression of key genes in *Bcl11b^−/−^* mice were qualitatively similar and characterized by down-regulation on the labial side and up-regulation on the lingual aspect of the developing incisor. The single exception to this observation was *Fst*, the expression domain of which appeared to be maintained by BCL11B. Collectively, these data suggest that BCL11B regulates the expression of downstream signaling molecules and transcription factors bidirectionally, activating expression on the labial side while repressing expression of the same genes on the lingual side (Fig. S13).

### Integration of BCL11B into FGF and SHH Signaling Pathways

**Figure 10:**
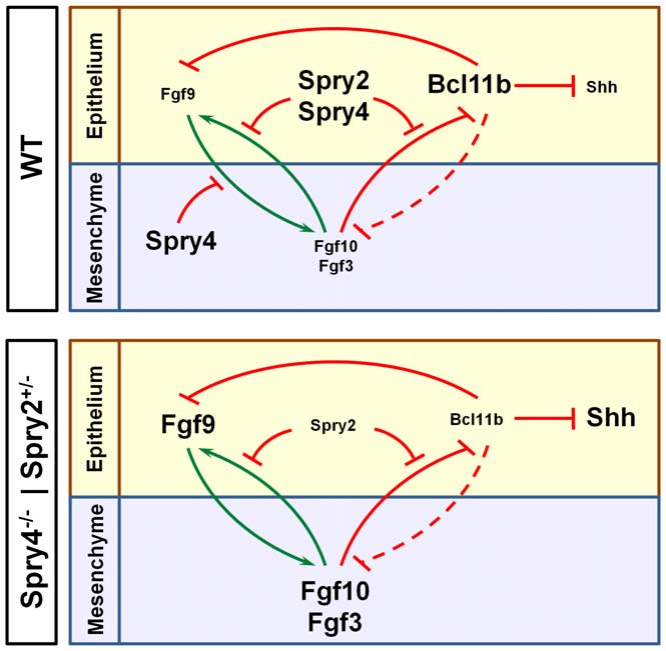
Model for a reciprocal, inhibitory circuit on the lingual side of the mouse incisor. (A) Proposed model for integration of BCL11B into FGF signaling pathways in the lingual epithelium of wild-type incisors. This model suggests that BCL11B expression in the lingual epithelium is facilitated by SPRY2- and SPRY4-mediated inhibition of the FGF signaling pathways. The resulting, high levels of BCL11B directly or indirectly suppress expression of *Shh* and subsequently lead to disruption of pre-ameloblast differentiation and ameloblast formation. It is also proposed that BCL11B inhibits expression of *Fgf9* in the lingual epithelium, and this serves to enforce disruption of the FGF signaling loop that is mediated by the Sprouty proteins. The repressive effect of BCL11B on *Fgf3* and *Fgf10* expression is most likely a secondary effect and is depicted by a dashed line. Therefore, BCL11B participates in the suppression of both FGF-mediated stimulation of lingual EpSC proliferation and differentiation of EpSC into *Shh*-positive pre-ameloblasts within the lingual IEE. (B) In the absence of *Spry4* and one allele of *Spry2*, FGF signaling is enhanced, which extinguishes expression of BCL11B in the lingual IEE. Loss of BCL11B results in derepression of *Fgf9* and *Shh* expression in the epithelium. FGF9 drives *Fgf3* and *Fgf10* expression in the lingual mesenchyme, whereas *Shh* expression is required for development of ectopic pre-ameloblasts within the lingual IEE.

Combined deletion of *Spry4* and one allele of *Spry2* [6] also results in ectopic development of *Shh*-positive pre-ameloblasts along the lingual epithelium of the developing incisor, suggesting a possible convergence in the FGF signaling pathways and BCL11B-dependent transcriptional regulation. This interpretation was supported by the strongly decreased expression of BCL11B within the lingual IEE of *Spry4^−/−^; Spry2^+/−^* incisors, indicating that unrestrained activity of the FGF signaling pathways results in pronounced down-regulation of BCL11B expression in the lingual IEE and subsequent de-repression of *Shh* expression. Based on these findings, we propose a model (Fig. 10) to explain the role of BCL11B in the FGF signaling pathways within the lingual epithelium at E16.5 (Fig. 10A) and in the *Spry4^−/−^; Spry2^+/−^* incisor (Fig. 10B). This model posits that:

1. SPRY4 and SPRY2 inhibit establishment of a FGF positive-feedback signaling loop [6] and the repressive effect of FGFs on BCL11B expression in the wild-type lingual IEE.
2. As a result, BCL11B is highly expressed in the entire lingual epithelium in wild-type mice and directly or indirectly inhibits *Fgf9* and *Shh* expression in the lingual epithelium, as was demonstrated in both *Bcl11b^−/−^* and *Bcl11b^ep−/−^* incisors. FGF9 acts in the mesenchyme to induce expression of *Fgf3* and *Fgf10* [6], [28]. Therefore, the repression of *Fgf9* expression by BCL11B in the lingual epithelium prevents activation of *Fgf3* and *Fgf10* expression in the dental mesenchyme. (Fig. 10A).
3. In contrast, Sprouty gene inactivation leads to up-regulation of FGF gene expression on the lingual side [6] and subsequent repression of BCL11B expression in the lingual IEE. This down-regulation of BCL11B expression leads to up-regulation of FGF family members, as well as that of *Shh*, which induces development of ectopic pre-ameloblasts in the lingual epithelium [7], [42] (Fig. 10B).

These data, which suggest that FGFs and BCL11B form a reciprocal, inhibitory circuit that is upstream of SHH, integrate FGFs, BCL11B, and SHH in a single pathway and provide insight into the molecular mechanisms underlying both the Sprouty and *Bcl11b^−/−^* phenotypes.

## Supporting Information

Figure S1 **Expression of** ***Bcl11b*** **at early bell stage.** (A) RNA ISH using *Bcl11b* probe in sections of wild-type mice at E16.5. (B-D) Sections of wild-type mice stained with DAPI and immunostained for BCL11B. Scale bar, 500 µm.
(TIF)

Figure S2 **Expression patterns of selected genes in** ***Bcl11b^−/−^*** **incisor at early bud stage.** RNA ISH using the indicated probes in sections of wild-type and *Bcl11b^−/−^* mice at E12.5. The epithelium is outlined by red dots. Scale bar, 100 µm.
(TIF)

Figure S3 **Expression patterns of selected genes in** ***Bcl11b^−/−^*** **incisor at cap stage.** RNA ISH using the indicated probes in sections of wild-type and *Bcl11b^−/−^* mice at E14.5. The epithelium is outlined by red dots. Scale bar, 200 µm.
(TIF)

Figure S4 **Delay in the initiation of apoptosis in** ***Bcl11b^−/−^*** **enamel knot at cap stage.** TUNEL immunostaining in sections of wild-type and *Bcl11b^−/−^* mice at indicated stages. The epithelium is outlined by white dots. White arrows denote apoptosis in the enamel knot. Scale bars: (A-B) 200 µm; other panels, 500 µm.
(TIF)

Figure S5 **Size difference between wild-type and** ***Bcl11b^−/−^*** **incisors of newborn mice.** Alizarin red staining of wild-type and *Bcl11b^−/−^* mandibular incisors at P0. Blue brackets indicate the posterior end of the incisor. Scale bar, 500 µm.
(TIF)

Figure S6 **Labial to lingual reversal of expression of** ***Gli1*** **in** ***Bcl11b^−/−^*** **incisors.** RNA ISH using a *Gli1* probe in sections of wild-type and *Bcl11b^−/−^* mice at indicated stages. The epithelium is outlined by red dots. Black and red arrows denote labial mesenchymal and epithelial staining, respectively, and red arrowheads indicate lingual epithelial staining. Scale bars: (A-B) 500 µm; other panels, 200 µm.
(TIF)

Figure S7 **Expression pattern of** ***Lfrn*** **and** ***Notch1*** **in** ***Bcl11b^−/−^*** **incisors.** RNA ISH using the indicated probes in sections of wild-type and *Bcl11b^−/−^* mice at indicated stages. The epithelium is outlined by red dots. Red arrows and arrowheads denote labial and lingual epithelial staining, respectively. Scale bars: (A-B, E-F) 500 µm; other panels, 200 µm.
(TIF)

Figure S8 **Expression of ameloblast markers in** ***Bcl11b^ep−/−^*** **and** ***Bcl11b^mes/−^*** **incisors at E18.5.** RNA ISH using the indicated probes in sections of *Bcl11b^L2/L2^*, *Bcl11b^ep−/−^*, and *Bcl11b^mes−/−^* mice at E18.5. The epithelium is outlined by red dots. Red arrowheads denote lingual epithelial staining. Scale bar, 200 µm.
(TIF)

Figure S9 **Morphology and mineralization of** ***Bcl11b^ep−/−^*** **incisors at P21.** Micro-CT analysis of Bcl11b^L2/L2^ and Bcl11b^ep−/−^ jaws at P21.
(TIF)

Figure S10 **Expression patterns of ameloblast markers and signaling molecules in** ***Bcl11b^mes/−^*** **incisors at E16.5.** (A-B) BCL11B immunostaining (red) in sections of *Bcl11b^L2/L2^* and *Bcl11b^mes−/−^* mice at E16.5. The epithelium is outlined by white dots. White asterisks denote BCL11B staining in the posterior mesenchyme. (C-J) RNA ISH using the indicated probes in sections of *Bcl11b^L2/L2^* and *Bcl11b^mes−/−^* mice at E16.5. The epithelium is outlined by red dots. Scale bar, 500 µm.
(TIF)

Figure S11 **Labial to lingual reversal of expression of** ***Tbx1*** **in** ***Bcl11b^−/−^*** **incisors.** RNA ISH using a *Tbx1* probe in sections of wild-type and *Bcl11b^−/−^* mice at indicated stages. The epithelium is outlined by red dots. Red arrows and arrowheads denote labial and lingual epithelial staining, respectively. Scale bars: (A-B) 500 µm; other panels, 200 µm.
(TIF)

Figure S12 **BCL11B expression in** ***Fgf3^−/−^; Fgf10^+/−^*** **incisors.** BCL11B immunostaining in sections of wild-type and *Fgf3^−/−^; Fgf10^+/−^* mice at E16.5. The epithelium is outlined by white dots. Scale bar, 500 µm.
(TIF)

Figure S13 **Summary of direct or indirect BCL11B target genes at E16.5.** This model is based on RNA ISH studies presented in Figs. 5,6,7 and Suppl. Figs. S5, S6, and S10. The red staining of the incisor is a pseudo-color representation of BCL11B immunohistochemical staining experiment. The epithelium is outlined by black dots. Green and red arrows indicate induction and inhibition of gene expression, respectively; blue dots denote the enforcement of gene expression domains by BCL11B.
(TIF)
