# Supplementary figures and images for: BCL11B Regulates Epithelial Proliferation and Asymmetric Development of the Mouse Mandibular Incisor
Source: PLoS One. 2012 May 22;7(5):e37670. doi: 10.1371/journal.pone.0037670 (PMC3358280; doi:10.1371/journal.pone.0037670)

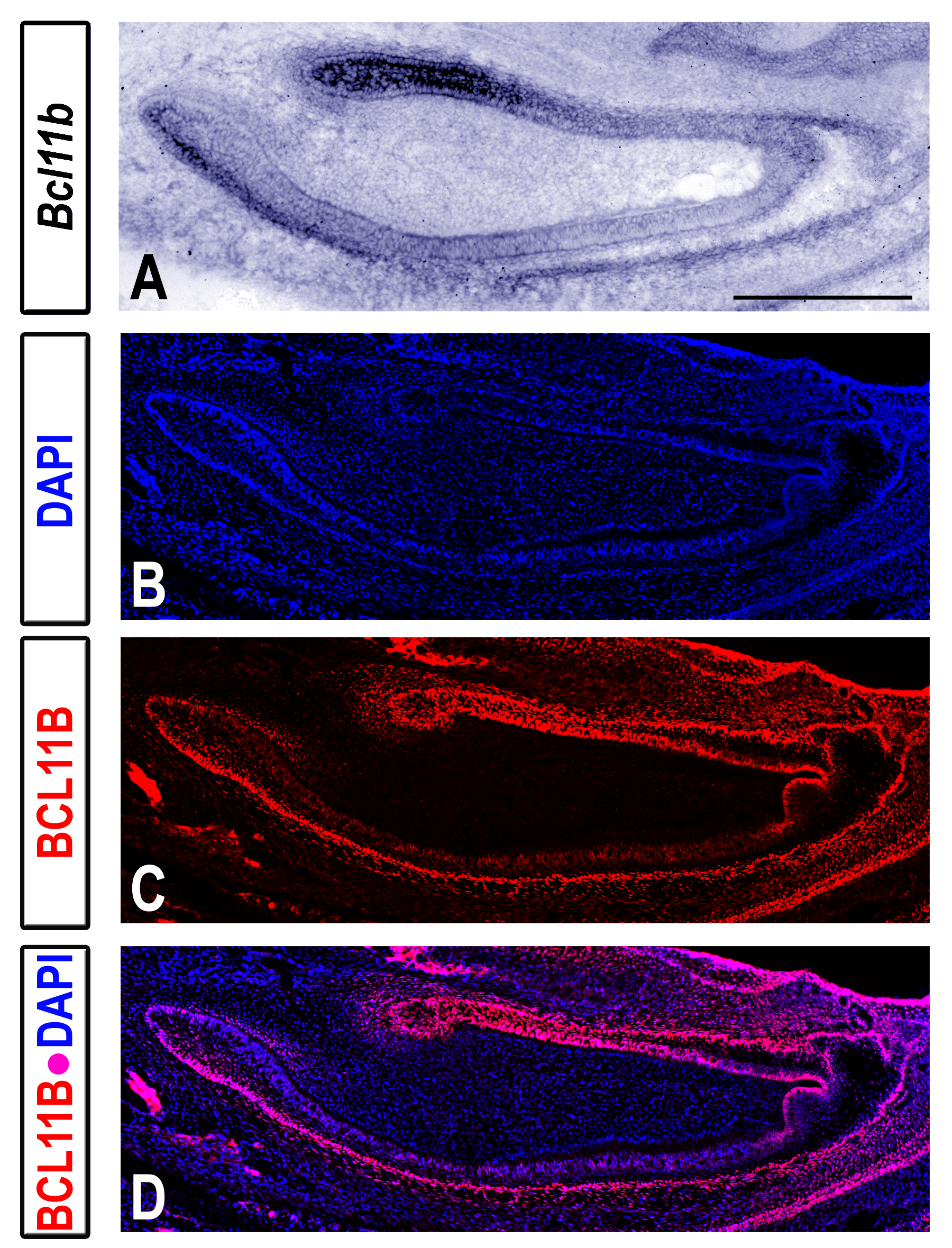

Supplement: Figure S1 — Expression of Bcl11b at early bell stage. (A) RNA ISH using Bcl11b probe in sections of wild-type mice at E16.5. (B-D) Sections of wild-type mice stained with DAPI and immunostained for BCL11B. Scale bar, 500 µm. (TIF) [file pone.0037670.s001.tif]

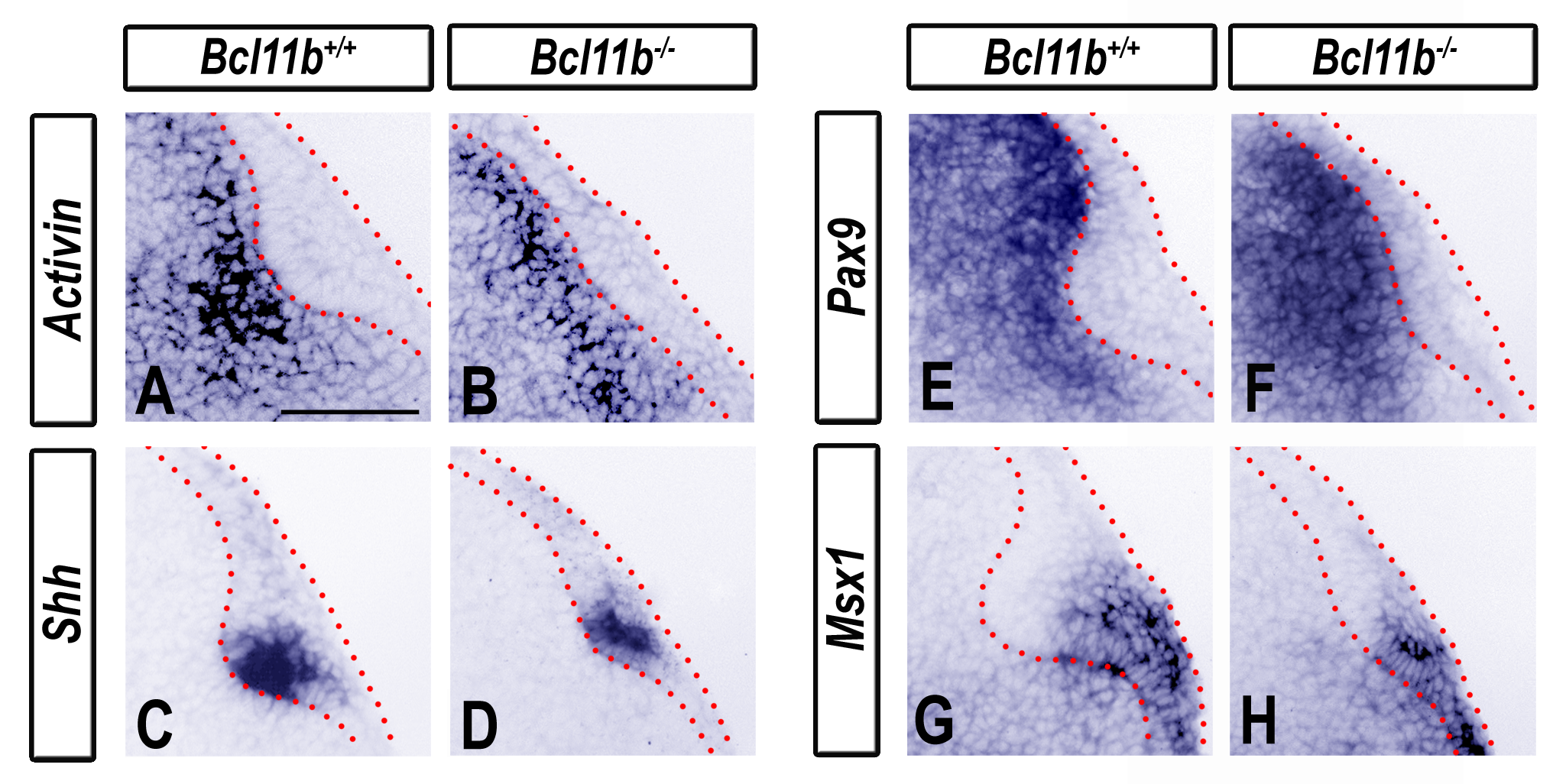

Supplement: Figure S2 — Expression patterns of selected genes in Bcl11b−/− incisor at early bud stage. RNA ISH using the indicated probes in sections of wild-type and Bcl11b−/− mice at E12.5. The epithelium is outlined by red dots. Scale bar, 100 µm. (TIF) [file pone.0037670.s002.tif]

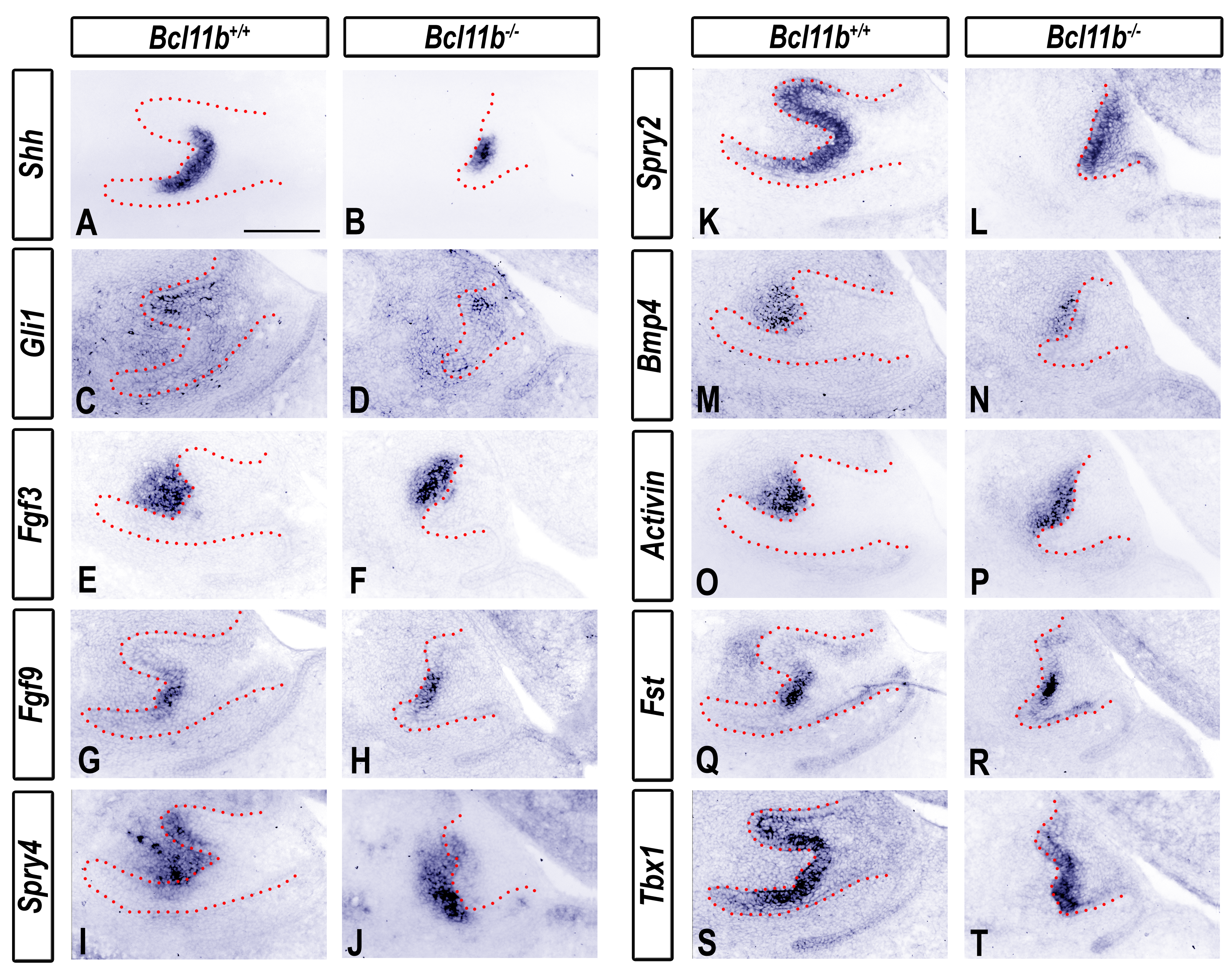

Supplement: Figure S3 — Expression patterns of selected genes in Bcl11b−/− incisor at cap stage. RNA ISH using the indicated probes in sections of wild-type and Bcl11b−/− mice at E14.5. The epithelium is outlined by red dots. Scale bar, 200 µm. (TIF) [file pone.0037670.s003.tif]

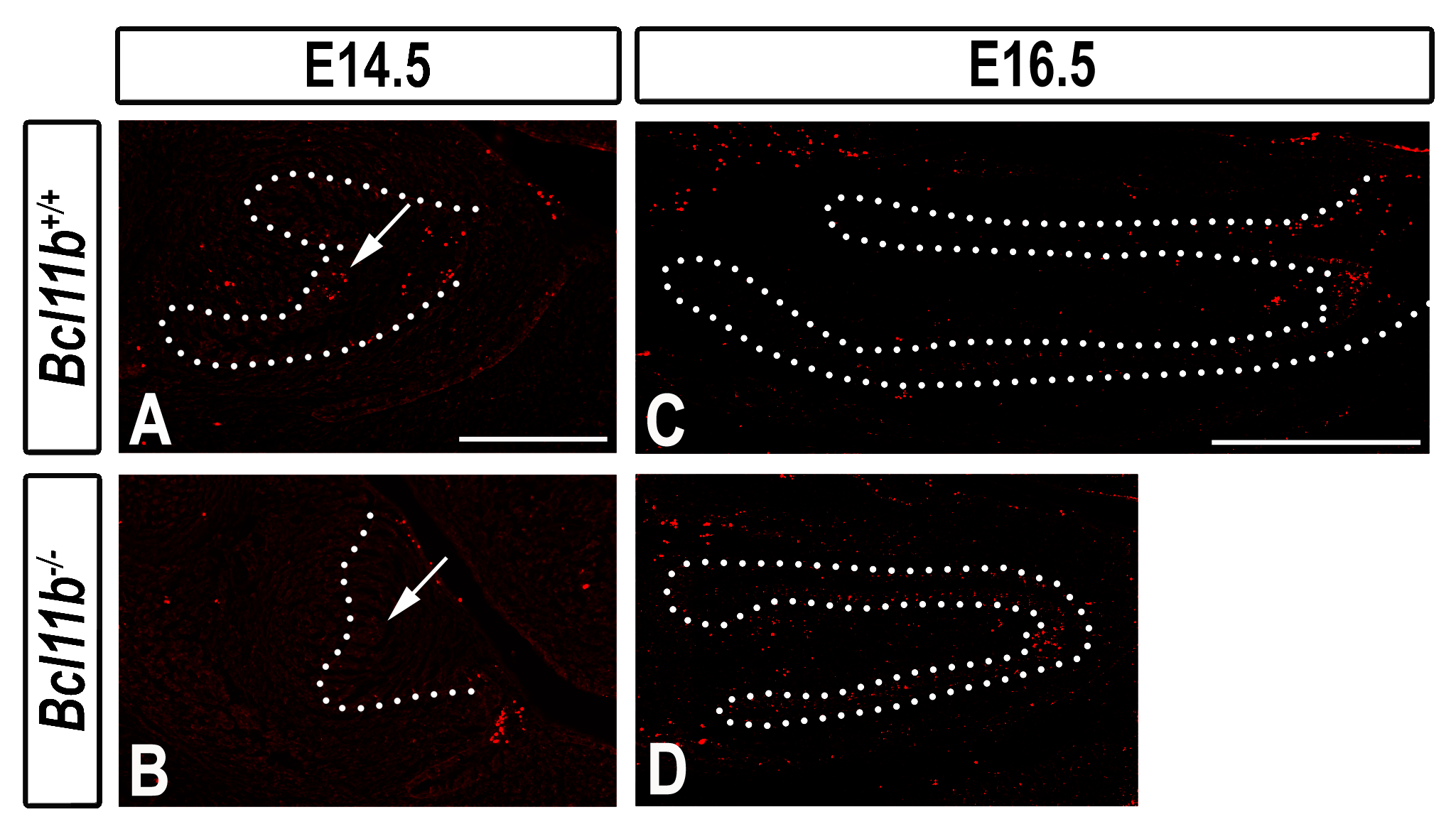

Supplement: Figure S4 — Delay in the initiation of apoptosis in Bcl11b−/− enamel knot at cap stage. TUNEL immunostaining in sections of wild-type and Bcl11b−/− mice at indicated stages. The epithelium is outlined by white dots. White arrows denote apoptosis in the enamel knot. Scale bars: (A-B) 200 µm; other panels, 500 µm. (TIF) [file pone.0037670.s004.tif]

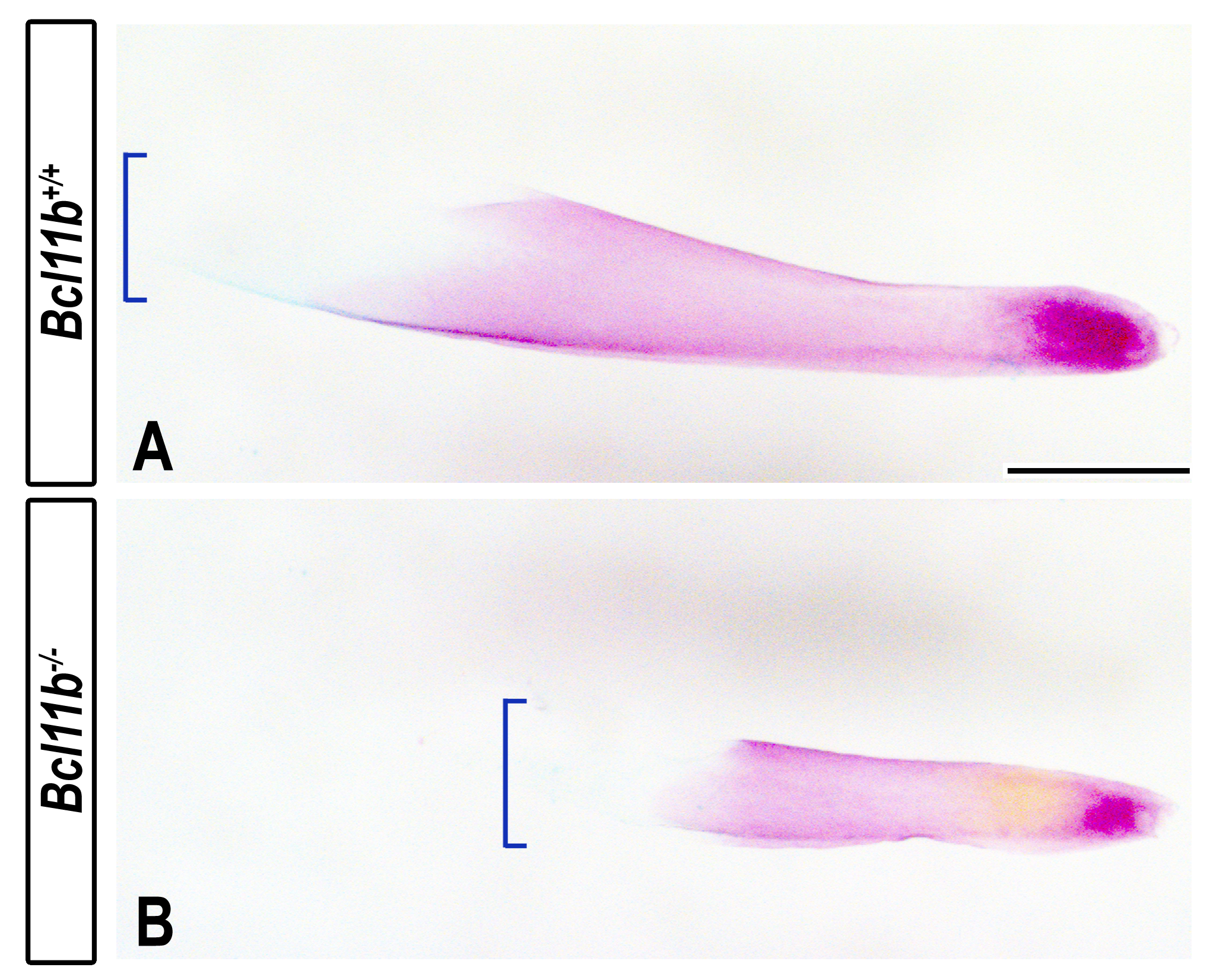

Supplement: Figure S5 — Size difference between wild-type and Bcl11b−/− incisors of newborn mice. Alizarin red staining of wild-type and Bcl11b−/− mandibular incisors at P0. Blue brackets indicate the posterior end of the incisor. Scale bar, 500 µm. (TIF) [file pone.0037670.s005.tif]

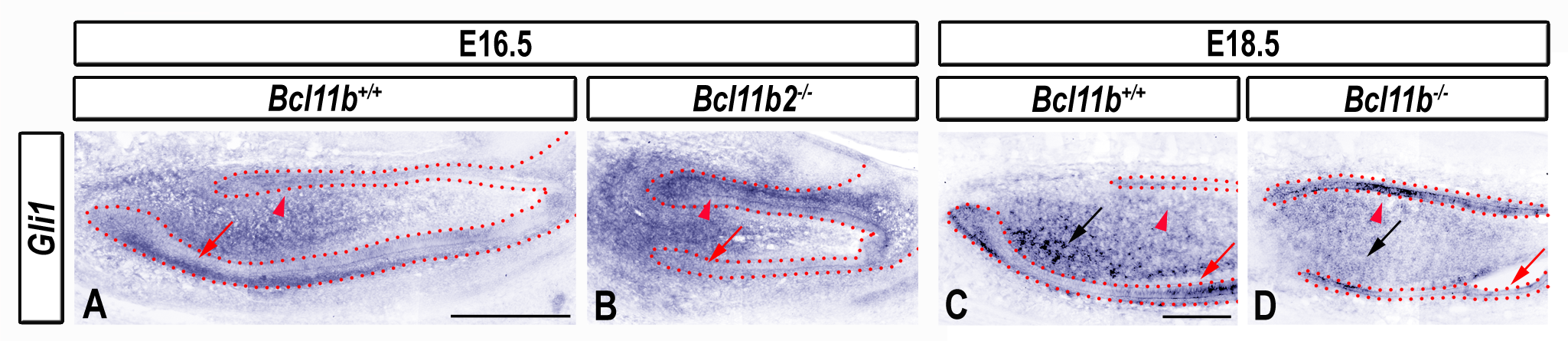

Supplement: Figure S6 — Labial to lingual reversal of expression of Gli1 in Bcl11b−/− incisors. RNA ISH using a Gli1 probe in sections of wild-type and Bcl11b−/− mice at indicated stages. The epithelium is outlined by red dots. Black and red arrows denote labial mesenchymal and epithelial staining, respectively, and red arrowheads indicate lingual epithelial staining. Scale bars: (A-B) 500 µm; other panels, 200 µm. (TIF) [file pone.0037670.s006.tif]

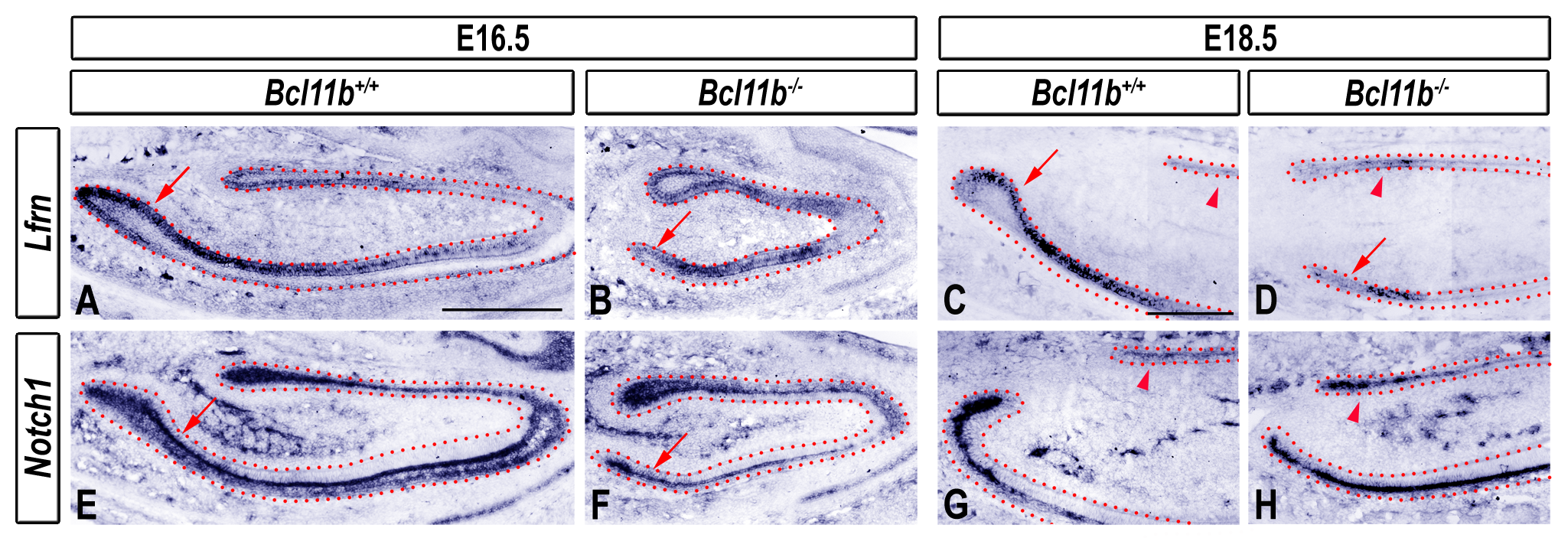

Supplement: Figure S7 — Expression pattern of Lfrn and Notch1 in Bcl11b−/− incisors. RNA ISH using the indicated probes in sections of wild-type and Bcl11b−/− mice at indicated stages. The epithelium is outlined by red dots. Red arrows and arrowheads denote labial and lingual epithelial staining, respectively. Scale bars: (A-B, E-F) 500 µm; other panels, 200 µm. (TIF) [file pone.0037670.s007.tif]

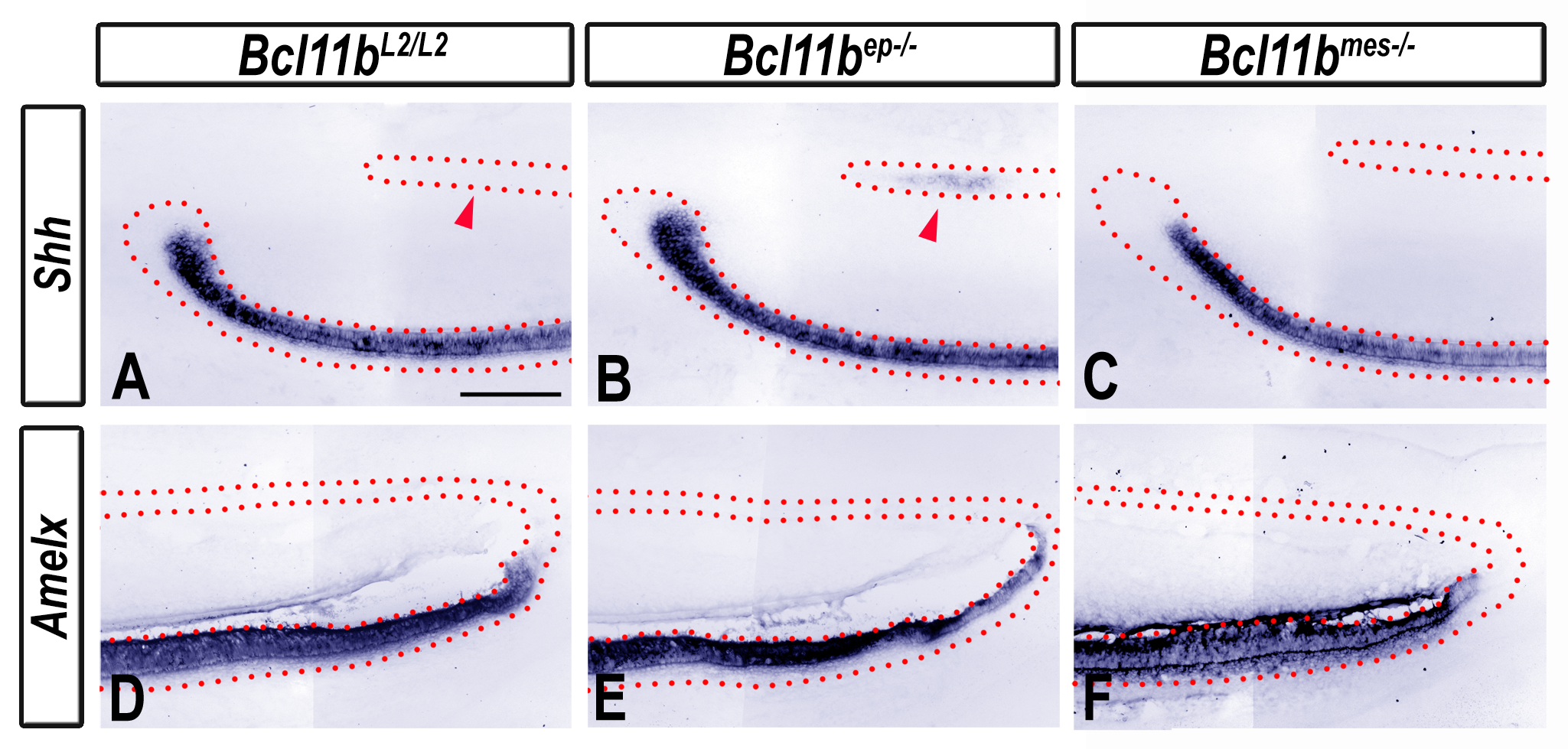

Supplement: Figure S8 — Expression of ameloblast markers in Bcl11bep−/− and Bcl11bmes/− incisors at E18.5. RNA ISH using the indicated probes in sections of Bcl11bL2/L2, Bcl11bep−/−, and Bcl11bmes−/− mice at E18.5. The epithelium is outlined by red dots. Red arrowheads denote lingual epithelial staining. Scale bar, 200 µm. (TIF) [file pone.0037670.s008.tif]

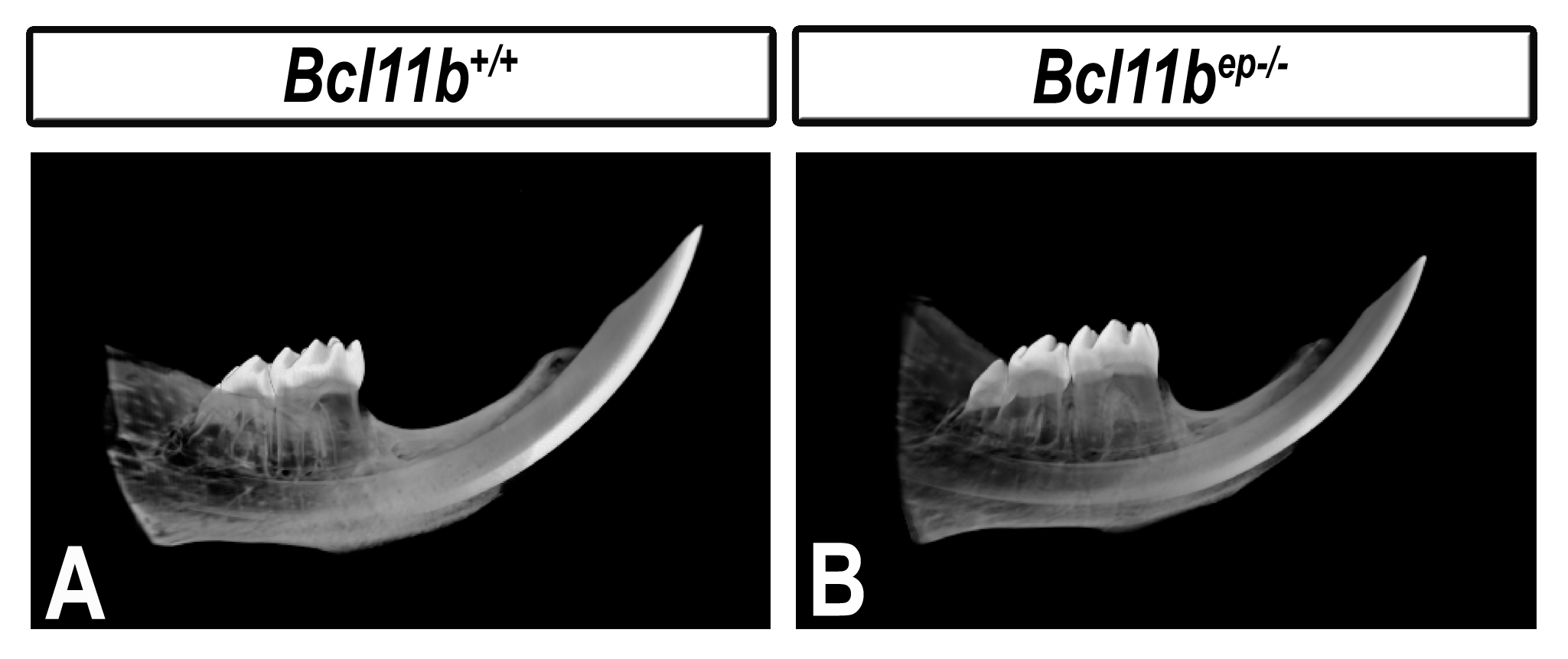

Supplement: Figure S9 — Morphology and mineralization of Bcl11bep−/− incisors at P21. Micro-CT analysis of Bcl11bL2/L2 and Bcl11bep−/− jaws at P21. (TIF) [file pone.0037670.s009.tif]

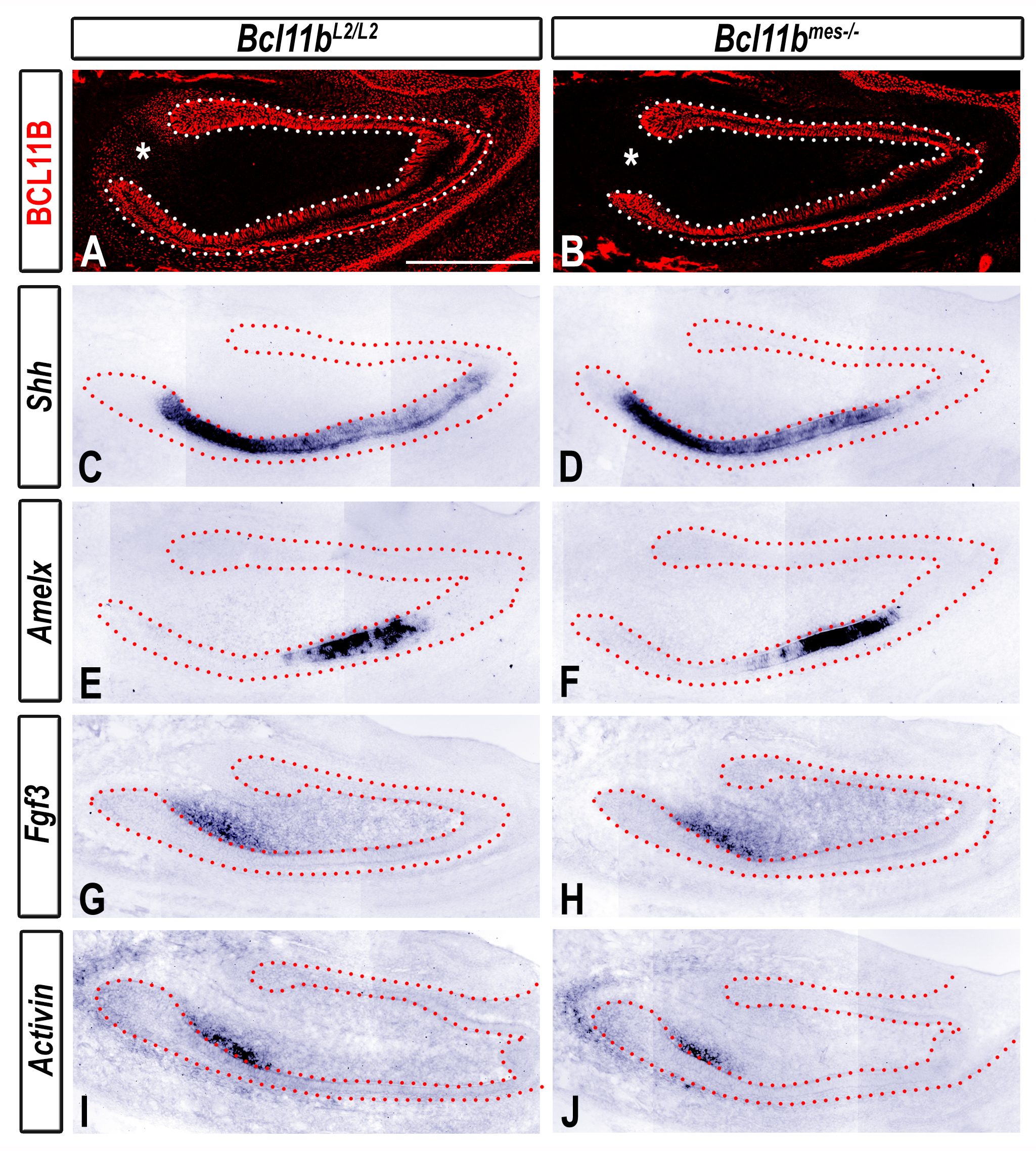

Supplement: Figure S10 — Expression patterns of ameloblast markers and signaling molecules in Bcl11bmes/− incisors at E16.5. (A-B) BCL11B immunostaining (red) in sections of Bcl11bL2/L2 and Bcl11bmes−/− mice at E16.5. The epithelium is outlined by white dots. White asterisks denote BCL11B staining in the posterior mesenchyme. (C-J) RNA ISH using the indicated probes in sections of Bcl11bL2/L2 and Bcl11bmes−/− mice at E16.5. The epithelium is outlined by red dots. Scale bar, 500 µm. (TIF) [file pone.0037670.s010.tif]

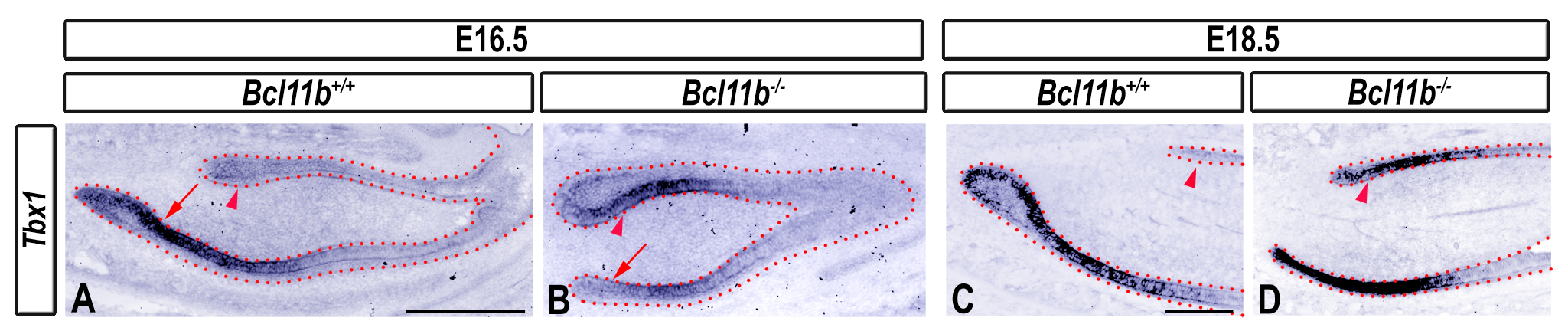

Supplement: Figure S11 — Labial to lingual reversal of expression of Tbx1 in Bcl11b−/− incisors. RNA ISH using a Tbx1 probe in sections of wild-type and Bcl11b−/− mice at indicated stages. The epithelium is outlined by red dots. Red arrows and arrowheads denote labial and lingual epithelial staining, respectively. Scale bars: (A-B) 500 µm; other panels, 200 µm. (TIF) [file pone.0037670.s011.tif]

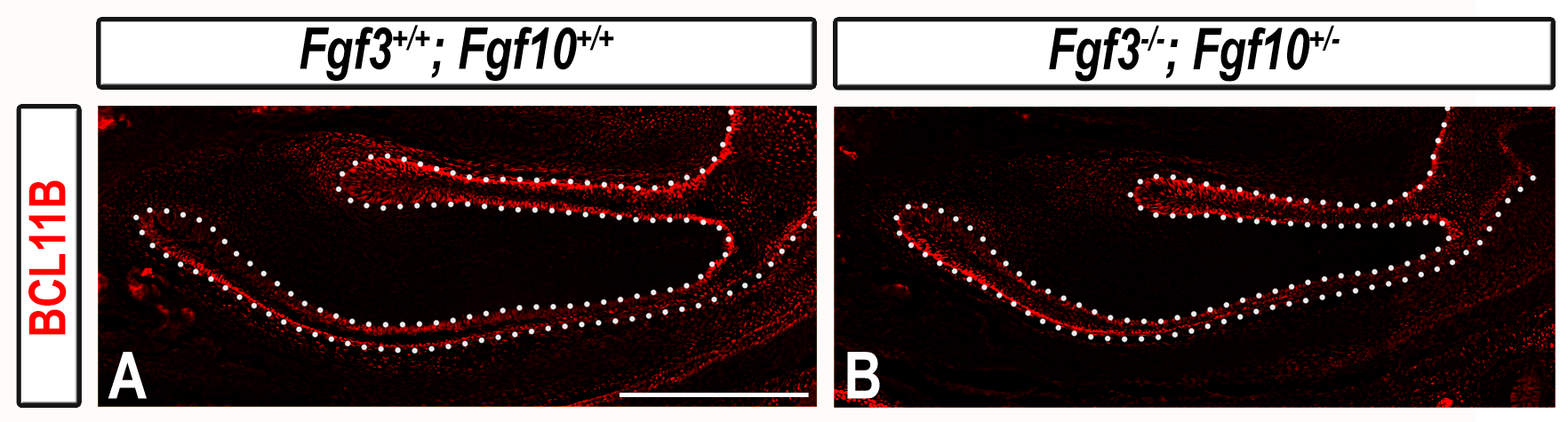

Supplement: Figure S12 — BCL11B expression in Fgf3−/−; Fgf10+/− incisors. BCL11B immunostaining in sections of wild-type and Fgf3−/−; Fgf10+/− mice at E16.5. The epithelium is outlined by white dots. Scale bar, 500 µm. (TIF) [file pone.0037670.s012.tif]

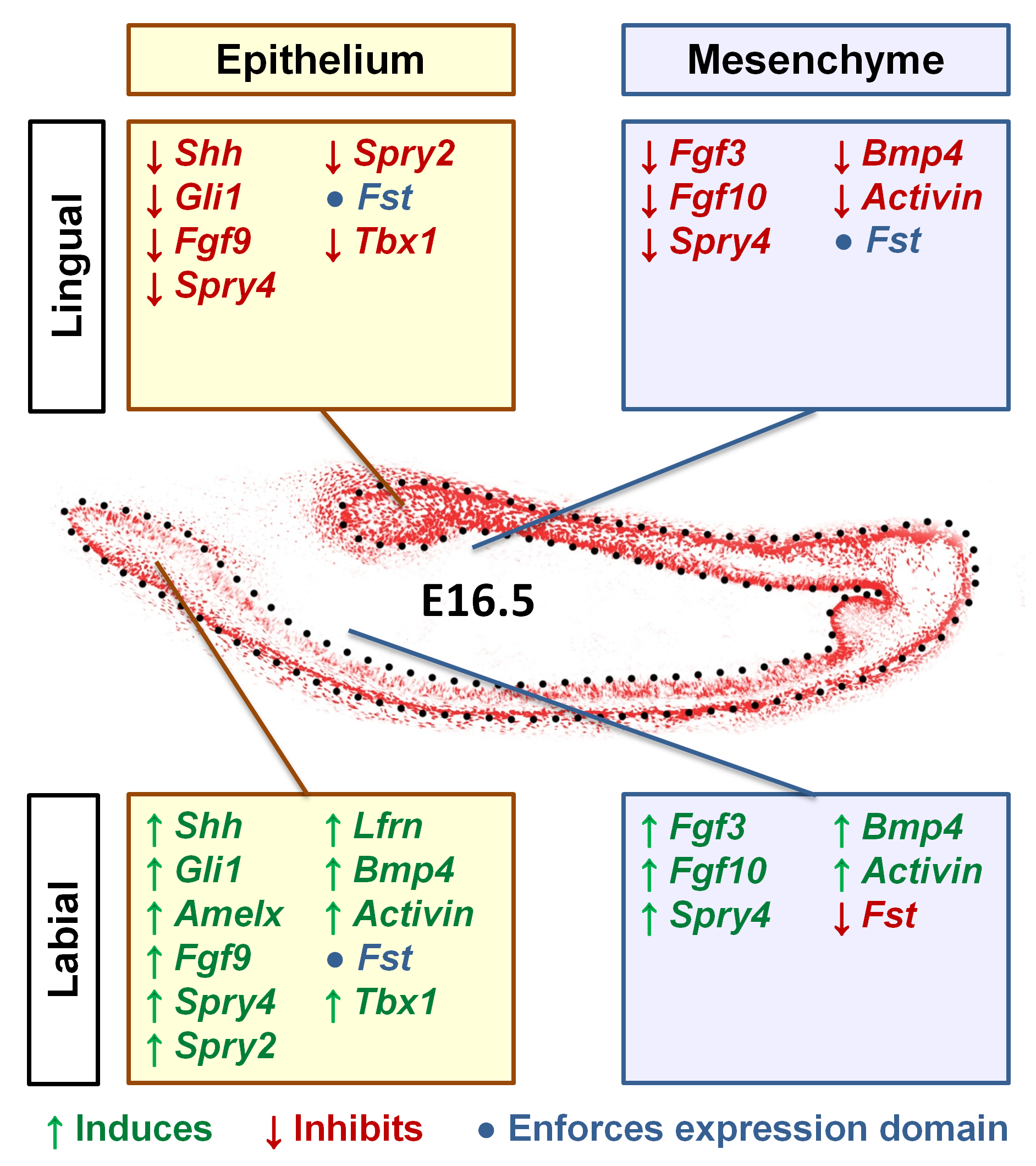

Supplement: Figure S13 — Summary of direct or indirect BCL11B target genes at E16.5. This model is based on RNA ISH studies presented in Figs. 5,6,7 and Suppl. Figs. S5, S6, and S10. The red staining of the incisor is a pseudo-color representation of BCL11B immunohistochemical staining experiment. The epithelium is outlined by black dots. Green and red arrows indicate induction and inhibition of gene expression, respectively; blue dots denote the enforcement of gene expression domains by BCL11B. (TIF) [file pone.0037670.s013.tif]
